# Translational behavioral phenotypes in DMSXL mice for CNS manifestations of DM1

**DOI:** 10.1177/22143602251410998

**Published:** 2026-01-14

**Authors:** Elisabetta Golini, Aline Huguet-Lachon, Hélène Benyamine, Noëmy Forasté Gueriba, Ferdinando Scavizzi, Marcello Raspa, Germana Falcone, Beatrice Cardinali, Silvia Mandillo, Geneviève Gourdon

**Affiliations:** 1 National Research Council (CNR), Institute of Biochemistry and Cell Biology (IBBC), Monterotondo (Rome), Italy; 2 National Research Council, CNR-EMMA-INFRAFRONTIER-IMPC, Monterotondo (Rome), Italy; 3 Sorbonne Université, Inserm, Institut de myologie, Centre de Recherche en Myologie, Paris, France; 4 Inserm, 27063 Institut Imagine, Paris, France

**Keywords:** myotonic dystrophy (DM1), DMSXL mouse model, behavioral phenotypes, cognitive dysfunction, emotional responses, social behavior

## Abstract

**Background:**

Myotonic dystrophy type 1 (DM1) is a multisystemic disorder frequently associated with central nervous system (CNS) involvement, especially in congenital and childhood-onset forms. However, behavioral alterations in preclinical models have so far been only partially characterized, underscoring the need for more comprehensive analyses to support the development of targeted therapeutic approaches.

**Objective:**

This study aimed to provide a comprehensive behavioral characterization of DMSXL mice, a transgenic model carrying large CTG repeat expansions in the human *DMPK* gene, to identify robust and translational CNS-relevant phenotypes for preclinical studies.

**Methods:**

Using both longitudinal and cross-sectional designs, we assessed a wide range of behavioral domains including motor function, emotional reactivity, cognition, and social interaction over time in DMSXL mice. The study was conducted in two independent laboratories with complementary expertise in DM1 pathophysiology and behavioral phenotyping.

**Results:**

DMSXL mice displayed a consistent pattern of behavioral alterations reflecting CNS dysfunction. These alterations included dysregulation of emotional responses such as altered anxiety-like behavior and impaired risk evaluation, subtle deficits in object recognition and spatial memory, and reduced sociability and social discrimination. Sensorimotor gating and goal-directed behaviors were also affected, while working memory and general locomotion during open field exploration were largely preserved.

**Conclusions:**

This study defines a constellation of behavioral impairments in DMSXL mice that mirror CNS symptoms in DM1 patients and establishes a set of sensitive, age-dependent endpoints suitable for CNS-targeted therapeutic evaluation. The behavioral framework presented here offers valuable guidance for the design of future preclinical trials in DM1.

## Introduction

Myotonic dystrophy type 1 (DM1) is an autosomal dominant multisystemic disorder,^1^ characterized not only by myotonia and progressive muscle weakness, but also by a broad range of clinical features, including cardiac conduction defects and central nervous system (CNS) alterations. DM1 is highly variable in terms of age at onset, symptom severity, and clinical presentation. Anticipation, manifested by increasing severity and earlier onset in successive generations, is particularly pronounced in DM1, which can affect adults, children, and newborns (congenital DM1, or CDM). Although initially considered primarily a muscle and cardiac disorder, accumulating clinical, neuropsychological, imaging, and histopathological evidence has demonstrated that DM1 is also a true brain disorder.^2–4^

Central nervous system (CNS) involvement in DM1 has been well documented in early-onset forms, especially congenital and pediatric-onset DM1. Although the congenital form (CDM) is associated with severe systemic symptoms at birth, children who survive the neonatal period frequently present with significant neurological and cognitive impairments. These include moderate to severe intellectual disability, reduced IQ, delays in speech and language development, deficits in visuospatial and visuo-constructive abilities, and attention deficit, with or without hyperactivity (ADHD). Several studies have also reported autism spectrum disorder, communication difficulties, and social anxiety.^5–11^ A parental survey further emphasized that communication difficulties and fatigue had the greatest impact on children's daily lives.^12^ In the pediatric form of DM1, classical neuromuscular symptoms may be absent. In such cases, cognitive impairments, such as visuo-spatial deficits, executive dysfunction, and learning difficulties, as well as psychiatric manifestations, including difficulties in forming relationships with peers, may represent the only clinical signs of the disease. Executive dysfunction, in particular, results in reduced initiative, impaired planning and decision-making abilities, and frequently leads to apathy and inactivity, which significantly impact quality of life in this patient population.

In adult DM1 patients, cognitive impairment with lower IQ scores is a common but variable feature, even in the least severe forms.^13^ Deficits in executive functions, episodic memory, and visuo-constructive abilities are frequently reported and are suggestive of a frontal dysexecutive syndrome.^7,^^14–16^ Patients are often described as apathetic, avoidant, and lacking initiative, with significant difficulties in social interactions and daily activities.^17^ Apathy has been linked to frontal lobe dysfunction and cognitive decline, independently of fatigue, sleepiness, age, or motor disability.^18^ Many DM1 patients also show impairments in social cognition, including difficulties recognizing facial expressions and understanding others’ mental states, as demonstrated by deficits in theory of mind tasks.^19–22^ In addition to cognitive and behavioral symptoms, DM1 patients frequently suffer from excessive daytime sleepiness (EDS), sleep apnea, periodic limb movements, REM sleep dysregulation, and prolonged nocturnal sleep.^23,24^ EDS appears to result primarily from central dysfunction of sleep regulation, while both respiratory muscle involvement and impaired central ventilatory control contribute to sleep-related breathing disorders.^25^ Fatigue, a major complaint in DM1, likely arises from both central and peripheral mechanisms, with CNS dysfunction playing a primary role in the subjective sense of tiredness.^26^

The identification in 1992 of the mutation responsible for DM1, located in the *DMPK* gene on chromosome 19, provided important clues to understanding this unusual pattern of inheritance. The underlying mutation is an abnormally expanded CTG trinucleotide repeat, which is highly unstable and typically increases in size across generations. This expansion is generally correlated with the severity of the disease.^27^ RNAs carrying expanded CUG repeats form intranuclear ribonuclear foci that accumulate in the nuclei of cells expressing the mutant *DMPK* gene during development and in various tissues. These foci interfere with a subset of RNA-binding proteins, including members of the MBNL and CELF families.^28,29^ These proteins are directly affected by the RNA foci either through sequestration, as in the case of MBNL proteins, or via post-translational stabilization, such as for CELF1. The resulting imbalance in the steady-state levels of these regulatory proteins disrupts multiple aspects of RNA metabolism, including transcription, alternative splicing, polyadenylation, mRNA stability, nuclear export, miRNA and RNA interference (RNAi) regulation, and both canonical and repeat-associated non-AUG (RAN) translation mechanisms. Toxic RNA foci have been observed in various brain regions and cell types, and numerous splicing defects have been reported.^30–35^ These findings suggest that RNA toxicity and its downstream consequences also contribute to central nervous system (CNS) dysfunction in DM1 patients. Among the RNA-binding proteins involved, MBNL2 appears to play a particularly important role in the CNS pathology of DM1.^36,37^ However, CELF proteins are also likely to be involved.^31,38,39^

To explore the molecular basis of CNS symptoms in DM1, several transgenic mouse models have been developed to gain insights into central nervous system (CNS) involvement in the disease. Knockout mice for MBNL1 and/or MBNL2 have confirmed the involvement of these proteins in CNS alterations. Loss of MBNL function induces splicing defects in the brain^33,^^40–44^ affecting GABAergic function,^45^ neuronal distribution, dendritic morphology, and postsynaptic architecture.^46,47^ These mice exhibit long-term cognitive deficits and a depressive-like state associated with neuronal loss, increased microglia, and decreased neurogenesis,^48,49^ as well as motivational deficits.^50^ Interestingly, they also show social deficits and autism-relevant mis-splicing profiles which are also observed in knockin mice with CTG expansions in the mouse gene.^51^ In order to study the contribution of large CTG repeats in the context of *DMPK* human sequences, we developed several years ago a transgenic mouse model carrying 45 kb of the DM locus with the *DMPK* gene and large CTG repeats.^52–54^ These mice express mutant *DMPK* RNA in various tissues, including the CNS, and display RNA foci in various brain regions associated with splicing defects.^52,^^54–56^ Foci are also observed in different brain cells, including neurons and astrocytes.^57,58^ Mice carrying >1000 CTG repeats show electrophysiological and behavioral deficits, such as novelty-induced inhibition, anhedonia, and excessive rest time.^38,59^ Reversal learning experiments also reveal differences in cognitive flexibility and motivation in DMSXL mice, reminiscent of what is observed in DM1 patients.^60^ They show deficits in short-term synaptic plasticity, as well as changes in neurochemistry and synaptic dysfunction.^38,61^ DMSXL mice are also used for preclinical studies aiming at targeting in various tissues the human *DMPK* sequences and the CTG repeat^62–69^ and CNS is of particular interest in this model that express significant levels of toxic *DMPK* mutant RNA in the brain.^54^ In this study, behavioral assessments were conducted in two independent and complementary laboratories. Longitudinal analyses were performed at the *Imagine* institute and at the Myology research center (CRM, Paris, France), in the laboratory where the DMSXL mouse model was originally developed and is extensively studied in the context of DM1. This time-course approach aimed to identify the temporal emergence of CNS-related phenotypes and define optimal windows for future preclinical therapeutic interventions. In parallel, in-depth behavioral investigations were carried out at the CNR Institute of Biochemistry and Cell Biology (CNR-IBBC, Rome, Italy), in the laboratory with recognized expertise in mouse behavioral phenotyping, to expand and refine the characterization of specific phenotypic traits. This cross-laboratory design reinforces the robustness and reproducibility of our findings and provides a comprehensive evaluation of CNS manifestations in DMSXL mice. Ultimately, these complementary strategies strengthen the translational relevance of the model and its potential use in preclinical testing of CNS-targeted therapies for myotonic dystrophy type 1.

## Material and methods

### Animal housing and genotyping of the French cohorts

Mice were housed in ventilated racks (Tecniplast GM500 cages, Buguggiate, Italy) under standard conditions with a 12-h light/dark cycle (lights on from 7:00 am to 7:00 pm), room temperature maintained between 22 °C and 24 °C, and relative humidity between 20% and 42%. Cages were changed every two weeks, with no cage changes during the week of behavioral testing. Animals had access to water via non-automated bottles. They were fed ad libitum with Safe A04 chow. Homozygous DMSXL mice (and tested WT controls) additionally received a dietary supplement consisting of diet gel and soft mash prepared from powdered chow mixed with water. Mice were housed and handled in accordance with European guidelines for the care and use of laboratory animals (Directive 86/609/EEC; French Decree 2001-131). The project was approved by the local ethics committee and the French Ministry of Higher Education, Research and Innovation (APAFIS#6418-201511101421606 v9 and APAFIS#23473-2019071014259655 v12).

DMSXL mice (C57BL/6 background) carry a 45 kb fragment of human genomic DNA containing the *DMPK* gene with an expanded CTG repeat (>1000CTG), as previously described.^53,70^ Genomic DNA was extracted from a tail biopsy collected at postnatal day 10. Genotyping was performed by PCR using primers specific to the Fbxl7 gene, where the transgene is integrated. The sequences of the oligonucleotide primers used (in a 1:0.5:0.5 ratio) were:

FBF: TCCTCAGAAGCACTCATCCG

FBWDR: ACCTCCATCCTTTCAGCACC

FBRBR: AACCCTGTATTTGACCCCAG

The CTG repeat length was verified by PCR, as previously described.^54^

### Behavioral testing in the French cohorts

**Figure 1.**
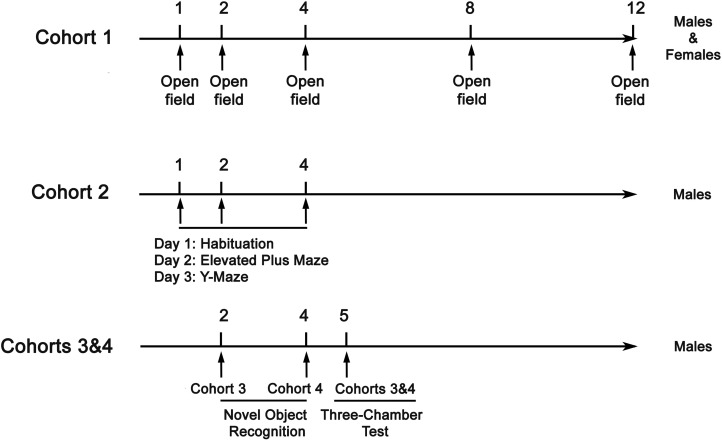
Experimental timeline of behavioral testing across three independent cohorts of DMSXL and WT mice in Paris, France. Cohort 1 included male and female mice (n = 16–18 per genotype and sex) tested longitudinally in the open field at 1, 2, 4, 8, and 12 months of age to assess locomotor activity, exploratory behavior, and anxiety-related responses. Cohort 2 consisted of male mice (n = 19–20 per genotype) tested at 1, 2, and 4 months of age. At each time point, animals underwent a 3-day testing sequence: day 1 – habituation for 15 min in the open field arena, day 2 – elevated plus maze, and day 3 – Y-maze, to evaluate anxiety-related behaviors and working memory. Cohorts 3 and 4 included male mice tested in the novel object recognition task at 2 months (Cohort 3, n = 8) and 4 months (Cohort 4, n = 8), and pooled for the three-chamber test conducted at 5 months of age (n = 16 per genotype), to assess recognition memory and social interaction behaviors, respectively.

All behavioral tests were conducted during the light phase (9 a.m. to 1 p.m.) using standardized protocols. All apparatus and devices were purchased from Bioseb (Vitrolles, France), unless otherwise specified. Mice were acclimated to the behavior room at least the day before testing by being placed on a dedicated rack in their home cages to allow habituation to the environment. DMSXL and WT mice were tested in alternation, and video analyses were performed blind to genotype. A detailed description of the cohorts used, as well as the timeline and distribution of the behavioral tests, is provided in Figure 1.

**Figure 2.**
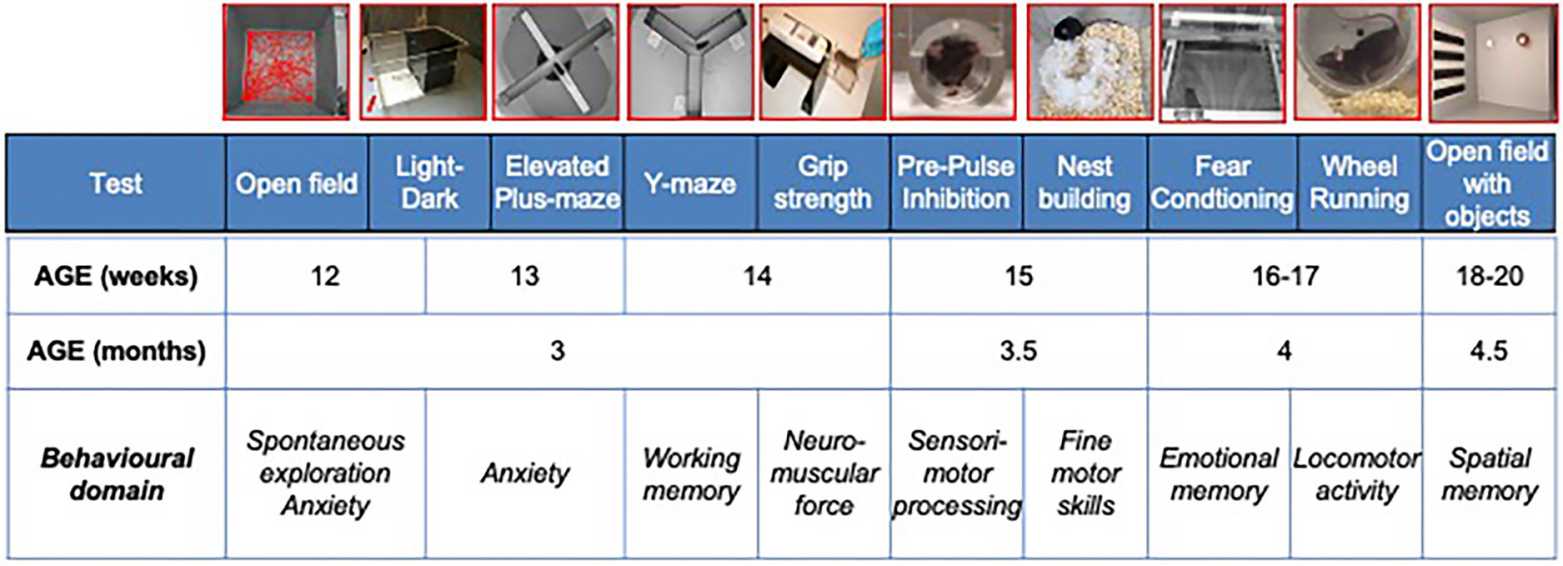
Experimental timeline at CNR-IBBC, Rome, Italy. Two cohorts of DMSXL mice and WT littermates between 12 and 20 weeks of age (3–5 months), were subjected to a comprehensive battery of behavioral tests assessing multiple functional domains. Tests were grouped by behavioral domain and administered in the order illustrated. Representative images are shown above each test. Testing was spaced to minimize stress and fatigue. Cohorts and group size are shown in Supplementary Table S1.

**Figure 3.**
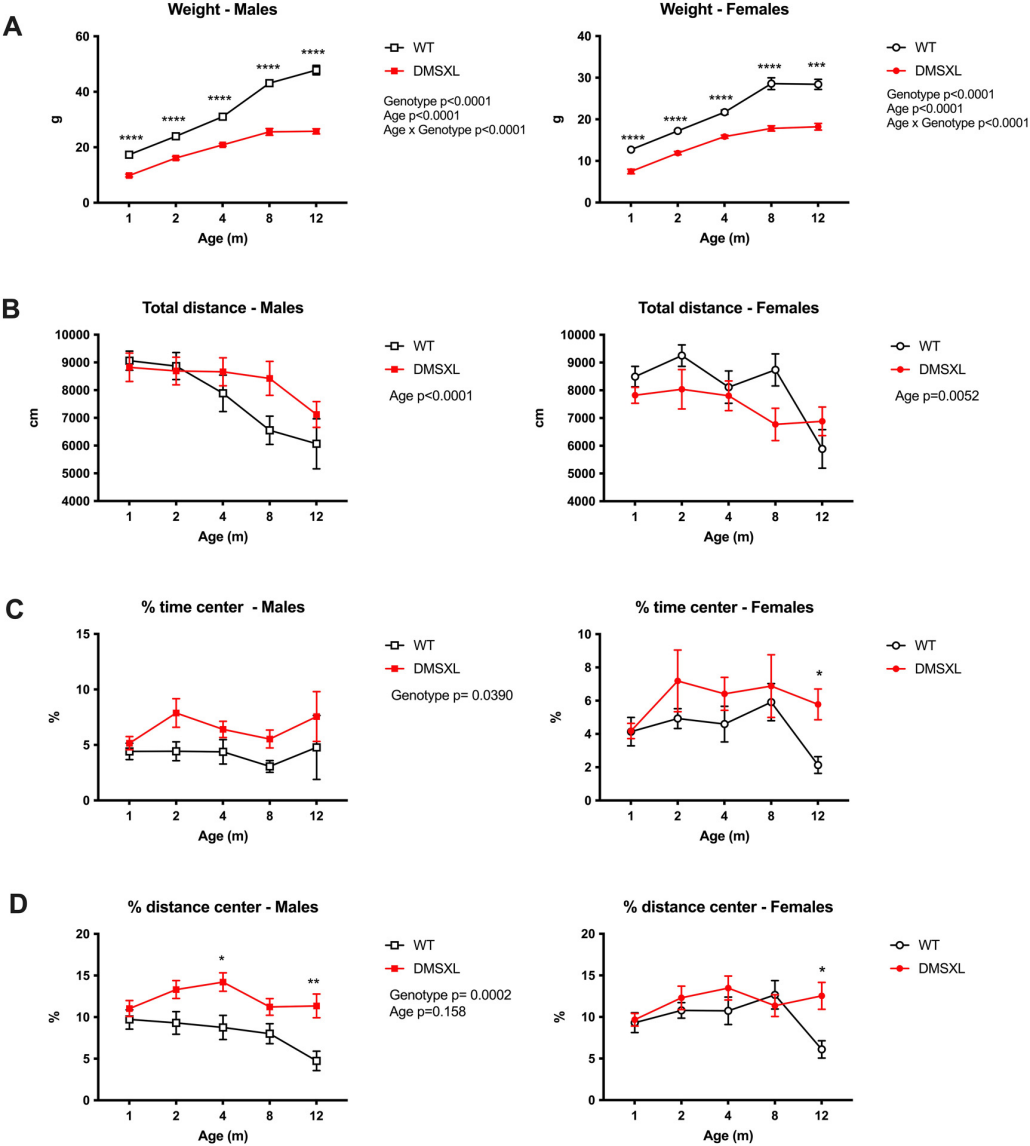
Longitudinal assessment of locomotor activity, and anxiety-like behavior in WT and DMSXL mice using the open field test. Male and female WT and DMSXL homozygous mice (n = 16–18 per group, except at 12 months for females where n = 6 per group) were tested at 1, 2, 4, 8, and 12 months of age. (A) DMSXL mice showed significantly lower body weight than WT littermates at all ages tested, consistent with previous reports. (B) Total distance traveled during the 30-min session declined with age in both genotypes and sexes. No significant genotype effect was detected, although DMSXL males tended to be slightly more active than WT at older ages. (C, D) Time spent and distance traveled in the central zone of the arena revealed a significant genotype effect in males, with DMSXL mice spending more time and traveling more distance in the center from 2 months of age onwards, suggesting reduced anxiety-like behavior or altered risk assessment. No significant differences were observed in females, except at 12 months, which should be interpreted cautiously due to smaller group sizes. Data are presented as mean ± SEM. Statistical analyses were performed using mixed-effects models followed by Sidak's multiple comparisons test. *p < 0.05, **p < 0.01.

**Figure 4.**
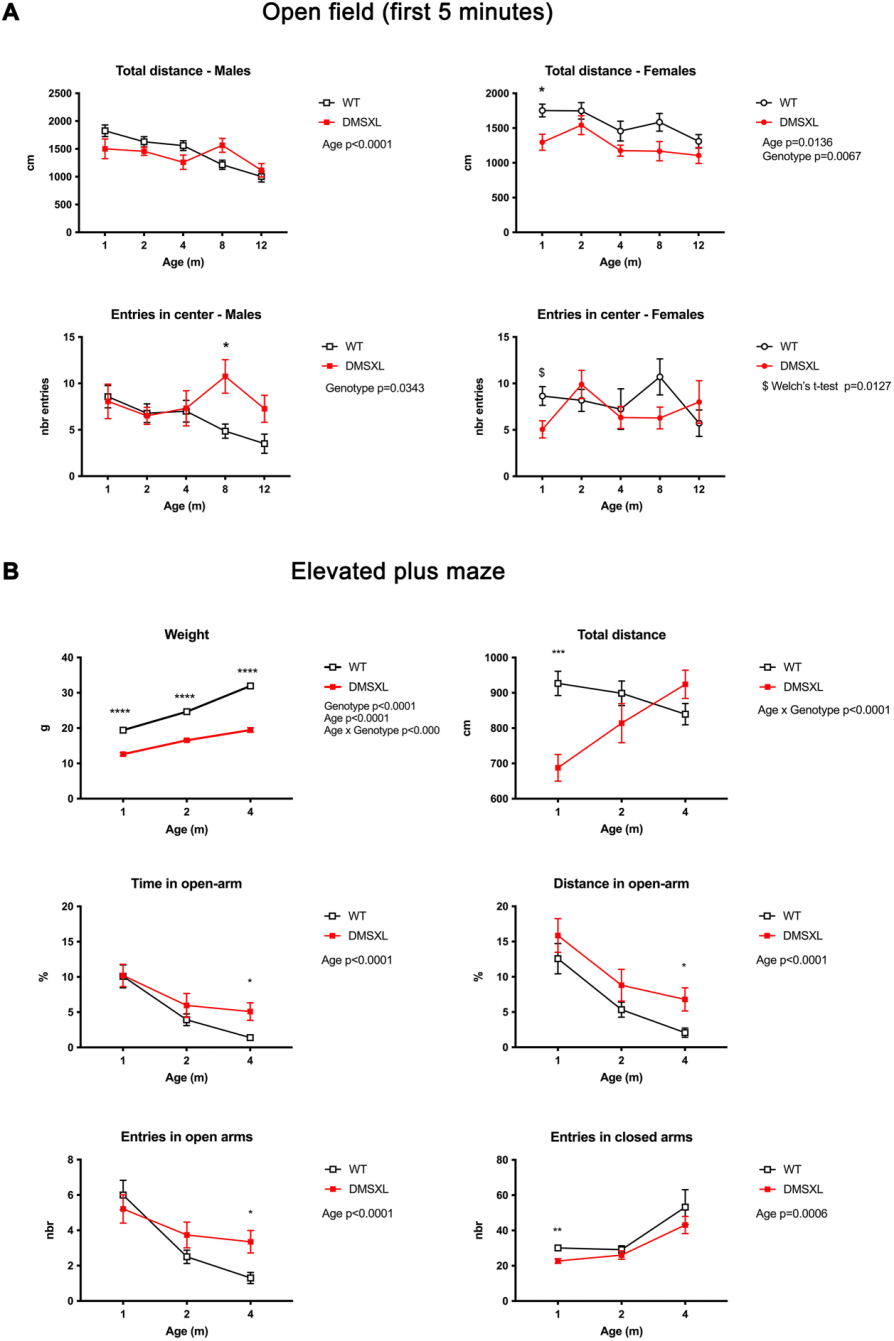
Exploratory and anxiety-like behaviors in WT and DMSXL mice: analysis of novelty-induced inhibition in the open field and performance in the elevated plus maze. (A) Open field. Male and female WT and homozygous DMSXL mice (n = 16–18 per group, except at 12 months for females where n = 6 per group) were tested longitudinally at 1, 2, 4, 8, and 12 months of age. In males, total distance traveled and number of entries into the center declined with age, with no significant genotype differences, although DMSXL mice showed a modest increase in center entries at 8 months. In females, a significant genotype effect was observed for total distance at 1 month, with DMSXL mice displaying reduced locomotion compared to WT. No significant main effects of age or genotype were detected for the number of center entries. However, a post hoc Welch's t- test revealed a significant reduction in the number of center entries in 1-month-old DMSXL females compared to WT, consistent with a transient novelty-induced inhibition phenotype no longer evident at later ages. (B) Elevated plus maze. Wild-type (WT) and homozygous DMSXL male mice (n = 16–20/genotype depending on age) were tested longitudinally at 1, 2, and 4 months of age in the elevated plus maze. Body weight was significantly reduced in DMSXL mice compared to WT at all ages tested. Total distance traveled showed a significant age × genotype interaction with reduced locomotor activity in DMSXL mice at 1 month, which normalized by 4 months. Anxiety-like behavior was assessed through the percentage of time and distance spent in the open arms. Both measures declined with age, consistent with increased avoidance of the open arms. At 4 months, DMSXL mice spent significantly more time and traveled a greater distance in the open arms compared to WT, suggesting altered risk assessment or disinhibition. DMSXL mice made significantly more entries into the open arms at 4 months than WT with a main effect of age on open-arm entries. In contrast, entries into the closed arms were significantly reduced in DMSXL mice at 1 month, reflecting early hypoactivity. Data are presented as mean ± SEM. Statistical analyses were performed using mixed-effects models followed by Sidak's multiple-comparisons test or Welch's *t*-test post hoc tests where indicated ($). *p* < 0.05, *p < 0.01, **p < 0.001, ***p < 0.0001.

**Figure 5.**
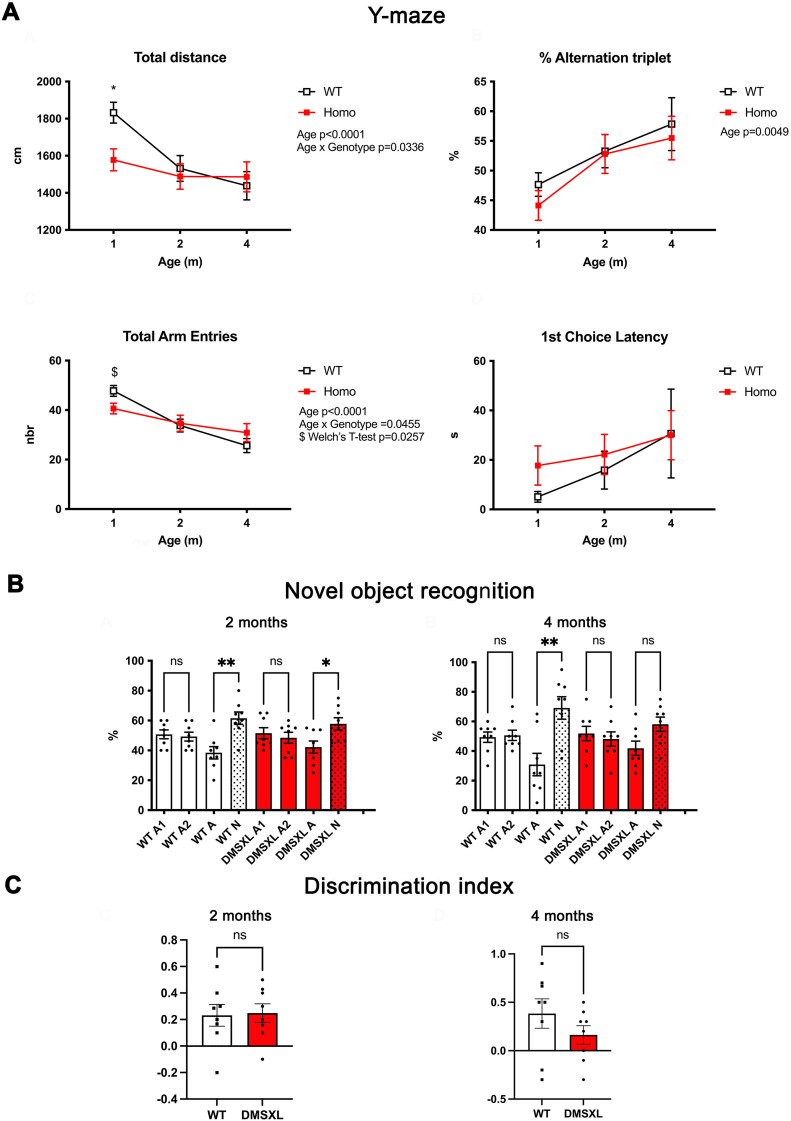
Exploratory activity, spatial working memory, and object recognition performance in WT and DMSXL male mice. (A) Y-maze spontaneous alternation test. Wild-type (WT) and homozygous DMSXL male mice (n = 17–20/genotype depending on age) were tested at 1, 2, and 4 months of age. At 1 month, DMSXL mice exhibited significantly reduced total distance traveled and total arm entries compared to WT littermates, indicating early hypoactivity. This difference was no longer detected at 2 and 4 months, suggesting normalization of exploratory behavior over time. The percentage of spontaneous alternations, a measure of spatial working memory, was similar between genotypes across all ages, indicating preserved working memory performance. Latency to the first arm entry did not differ between WT and DMSXL mice at any time point, suggesting intact initiation of exploration and absence of strong novelty-induced inhibition in this task. (B) Novel object recognition test (NOR). Long-term object recognition memory was evaluated in independent cohorts of male WT and DMSXL mice (n = 8 per group) at 2 and 4 months of age using a 24-h delay between familiarization and test phases. During the familiarization phase, mice spent equal amounts of time exploring the identical objects (A1, A2). At 2 months, both WT and DMSXL mice spent significantly more time exploring the novel object (N) compared to the familiar one (A) indicating preserved recognition memory at this age in both genotypes. At 4 months, WT mice maintained a significant preference for the novel object (N), while DMSXL mice exhibited a similar trend that did not reach significance, suggesting a weaker and less consistent novelty preference. (C) Discrimination index (DI): The DI did not reveal significant genotype differences, likely due to greater inter-individual variability and reduced total exploration time in some DMSXL mice. Data are presented as mean ± SEM. Statistical analyses were performed using mixed-effects models followed by Sidak's multiple-comparisons test (Y-maze), Kruskal–Wallis test followed by Dunn's post hoc test (NOR) or Mann–Whitney U test for genotype comparison of the DI. *p < 0.05, **p < 0.01.

**Figure 6.**
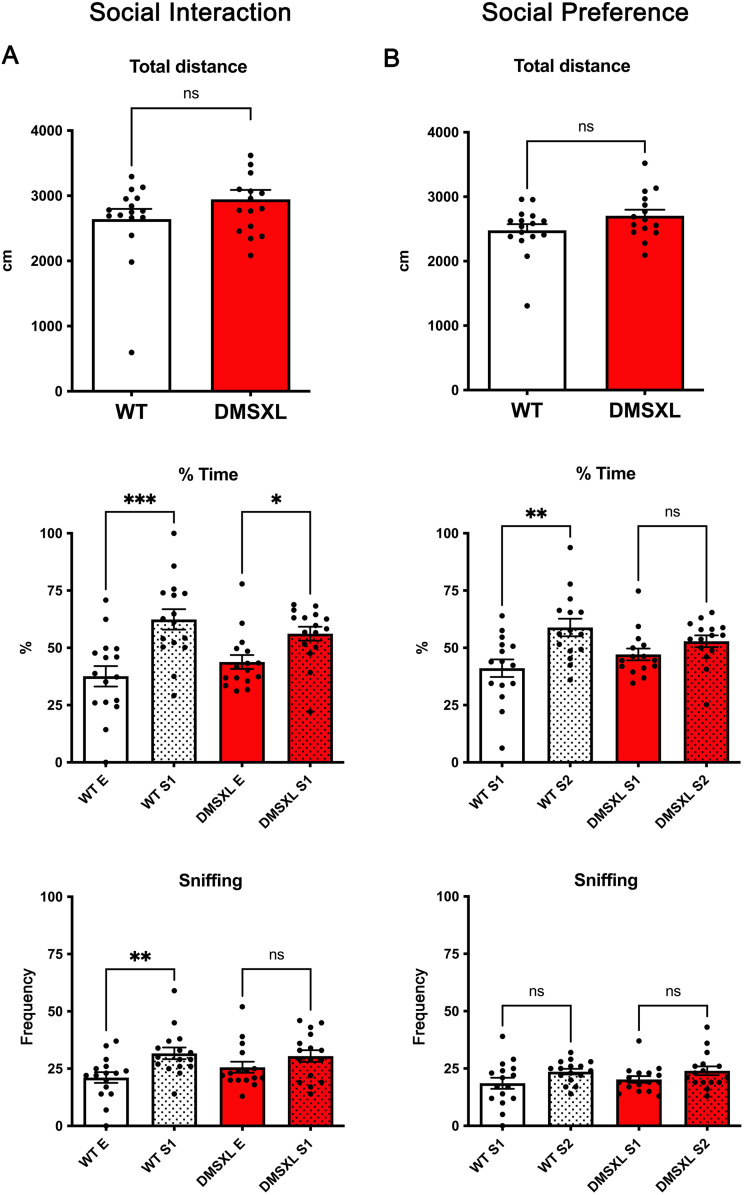
Social behavior assessment in the three-chamber test. Social interaction and social preference were evaluated at 5 months of age in male WT and DMSXL mice (n = 16/genotype). (A) Social interaction phase: Total distance traveled (top), percentage of time spent in each compartment (middle: Empty (E) vs. Stranger 1 (S1)), and frequency of sniffing bouts (bottom: E vs. S1). Both WT and DMSXL mice spent significantly more time in the compartment containing the unfamiliar mouse (S1) compared to the empty cage, indicating preserved sociability in both genotypes. However, this preference was more pronounced in WT mice. WT animals also displayed a significantly higher number of sniffing bouts toward S1, while this preference was not significant in DMSXL mice. (B) Social preference phase: Total distance traveled (top), percentage of time spent in each compartment (middle: Stranger 1 (S1) vs. Stranger 2 (S2)), and frequency of sniffing bouts (bottom: S1 vs. S2). WT mice showed a significant preference for the novel stranger (S2) over the familiar one (S1), whereas DMSXL mice did not exhibit a significant compartment preference. No significant difference in sniffing bouts between S1 and S2 was observed in either genotype, suggesting that this parameter may be less sensitive to detect social novelty in this setting. Together, these findings suggest that while WT mice exhibit intact sociability and social novelty recognition at 5 months, DMSXL mice show reduced social interaction drive and impaired social discrimination. Statistical analyses were performed using Kruskal–Wallis tests followed by Dunn's post hoc multiple comparisons. *p < 0.05, **p < 0.01, ***p < 0.001.

#### Open field

The open field consisted of a white, matte-finish plastic box (45 cm × 45 cm) with 40 cm high walls. The arena was monitored by a digital camera (DFX 22AUC03) connected to SMART v3.0 software. Animals were allowed to explore the arena for 30 min under controlled lighting conditions (150 lux along the walls and 180 lux in the center). Behavioral analysis was performed using SMART v3.0. Tests were conducted in the morning, and DMSXL and WT animals were assessed in an alternating order. After each session, the arena was cleaned using ethanol wipes, followed by thorough rinsing with water and drying.

#### Elevated plus maze

The elevated plus maze (EPM) consisted of two open arms and two enclosed arms (5 cm wide × 75 cm long), elevated 45 cm above the ground. The central zone was defined as a 5 cm × 5 cm square at the intersection of the arms. Mice were placed in the central zone at the beginning of the trial, and their position was tracked via a digital camera (DFX 22AUC03) connected to SMART v3.0 software (Panlab, Harvard Apparatus). Tests were conducted in the morning under controlled lighting conditions (180 lux in the open arms and 150 lux in the center). The mouse location was determined by the position of the body centroid. Each trial lasted 10 min. DMSXL and WT mice were tested in alternation, and video recordings were analyzed blind to genotype. After each session, the apparatus was cleaned with 70% ethanol and allowed to dry.

#### Y-maze

The Y-maze, made of gray polyvinyl chloride, was placed in a behavior room with uniform lighting (150 lux in each arm). The maze consisted of three arms (8 × 30 × 15 cm; width × length × height), arranged at 120° angles. Mice were placed at the center of the maze and allowed to freely explore for 8 min. All sessions were recorded using a digital camera (DFX 22AUC03) connected to SMART v3.0 software. We analyzed the number of entries and the time spent in each arm. Spontaneous alternation behavior was calculated as the number of triads of consecutive entries into all three arms divided by the total number of arm entries minus two. Between each trial, the maze was cleaned with 70% ethanol and allowed to dry.

#### Novel object recognition test

The novel object recognition test was conducted in a white, matte-finish plastic 45 cm × 45 cm box with 40 cm walls and monitored by a digital camera (DFX 22AUC03), connected to SMART v3.0 software. The test consisted of two 10 min phases: (1) exploration and (2) test. To reduce novelty-induced stress, mice were habituated to the empty arena for 30 min the day before testing. In the exploration phase, mice were placed in the arena containing two identical novel objects (randomly selected from a colored tower of Lego bricks or a filled flask, Supplementary Figure S1, both 4 × 10 cm^71^) and allowed to explore freely. Twenty-four hours later, in the test phase, mice were reintroduced into the same arena, now containing one familiar and one novel object placed in the same locations as during the exploration phase. The identity and position of the novel object were systematically alternated across animals and equally balanced between genotypes. The arena and objects were cleaned or replaced between animals to eliminate olfactory cues.

Object investigation was manually scored from video by two experimenters blinded to genotype. Only exploration bouts where the animal's nose was directed toward the object at close proximity were counted, using two separate stopwatches (one per object), until the total investigation time (novel + familiar objects) reached 20 s. For each mouse, the percentage of time spent investigating the novel and familiar objects was calculated based on this 20-s total. The discrimination index was calculated as (time spent exploring the novel object - time spent exploring the familiar object) / (time spent exploring both objects), providing a normalized measure of recognition memory performance.^72^

#### Three-chamber sociability test

The three-chamber sociability test was used to assess social preference and recognition. The testing apparatus consisted of a three-chambered rectangular box (20 × 42 × 22 cm, L × W × H), with dividing walls featuring small openings to allow free access between chambers. The two outer chambers contained wire cup-like cages (10 cm bottom diameter, 13 cm height), enabling auditory, olfactory, and visual interaction, but preventing physical contact (Supplementary Figure S2). Habituation procedures were carried out prior to testing: the test mouse was first habituated to the apparatus and the empty wire cups the day before the test (10 min), and again for 5 min on the test day. The stimulus mouse was also habituated to the wire cup-like cages the day before, in two sessions of 5 min each. The test comprised three consecutive phases:

1. **Habituation** (5 min): the test mouse was placed in the apparatus with both wire cups empty, to acclimate to the environment.
2. **Social interaction** (10 min): one wire cup contained an unfamiliar, age-matched, same-sex conspecific (Stranger 1), while the other remained empty.
3. **Social preference** (10 min): Stranger 1 remained in its cup, while a second novel conspecific (Stranger 2) was placed in the opposite cup.

At the beginning of each phase involving social stimuli, the stimulus mouse was first positioned inside the wire cup, and the test mouse was then placed in the central chamber with the side doors closed. All doors were then opened simultaneously to allow free exploration. The locations of the social stimuli (familiar vs. novel) were systematically alternated between animals to avoid spatial bias.

All sessions were recorded using a digital camera (DFX 22AUC03) connected to SMART v3.0 software, and the time spent in interaction with the wire cup-like cages was scored. After each run, the wire cups were cleaned with ethanol and left to dry, and the apparatus was cleaned with 70% ethanol and allowed to dry.

Social interaction and preference were quantified by measuring the percentage of time spent in each chamber and the frequency of direct sniffing bouts directed at the wire cups (number of bouts per 10 min session).

#### Statistical analysis

For all behavioral data, mixed-effects models were used to assess the effects of age and genotype, followed by Sidak's multiple comparisons test when appropriate. In addition, post hoc analyses were performed at individual time points to compare genotypes using the non-parametric Mann–Whitney U test as a complementary approach. For simple comparisons between genotypes on single behavioral parameters (e.g., total distance traveled, time spent in open arms, or alternation rates), data were assessed for normality and analyzed using unpaired t-tests or Mann–Whitney U tests as appropriate.

For behavioral paradigms involving multiple within-subject conditions (e.g., exploration of familiar vs. novel objects in the novel object recognition test, or empty cage vs. stranger mouse in the social interaction test), data were initially considered for matched-pair analysis. However, due to the presence of occasional missing values (e.g., one DMSXL mouse missing in the social preference phase), we opted for a non-parametric unpaired approach to preserve all available data and maintain consistency across cohorts. Consequently, group comparisons were performed using the Kruskal–Wallis test followed by Dunn's multiple comparisons correction.

All analyses were conducted using GraphPad Prism (version 10), with significance set at *p* < 0.05.

### Animals and housing of the Italian cohorts

A DMSXL mouse colony was established at the IBBC-EMMA-Infrafrontier animal facility (Monterotondo, Italy) by crossing hemizygous male and female mice obtained from G. Gourdon. Prior to mating, the CTG triplet expansion in each hemizygous animal was verified using long-range PCR and Southern blot analysis.^73^ Offspring were genotyped by PCR of tail samples DNA^56^ and sex matched litters were group housed (n = 3–5 mice of mixed genotypes) in standard IVC cages (Tecniplast S.p.A) with food and chlorinated, filtered water ad libitum. Room temperature was 21 ± 2°C, relative humidity was 50–60%, and mice were kept in a 12 h light/dark cycle with lights on at 07:00 am until 07:00 pm.

DMSXL homozygous mice required a special care during the first month after birth. As previously reported by Huguet et al.,^54^ these animals exhibited high perinatal mortality and significant postnatal growth delay (Supplementary Figure S3). To address these challenges, both breeding pairs and weaned litters were provided with an energy-rich diet (EMMA23; Mucedola, Settimo Milanese, Italy). In addition, gel-based nutritional supplements (DietGel76A; Clear H2O, Westbrook, ME) and moistened chow were placed on the floor of all cages to facilitate access for homozygous pups. Some homozygous mice also developed misaligned upper incisors, which required regular trimming to ensure adequate feeding. These welfare-related issues persisted throughout the entire duration of the study. Animals were subjected to an experimental protocol approved by the Veterinary Department of the Italian Ministry of Health (no. 832/2019-PR), and experiments were conducted according to the ethical and safety rules and guidelines for the use of animals in biomedical research provided by the relevant Italian laws and European Union's directives (no. 86/609/EEC and subsequent).

### Experimental design in Italian cohorts

A comprehensive battery of behavioral tests was conducted in DMSXL homozygous (DMSXL) mice and their age- and sex-matched wild-type (WT) littermates. Two independent cohorts were used (Table S1), comprising a total of 28 WT and 23 DMSXL mice. The first cohort included only male animals, as female littermates were destinated to pilot studies for a previously published work from our group.^66^ The second cohort included both sexes. Adult mice were assessed from 12 to 20 weeks of age (3–5 months) across a range of functional assays to evaluate spontaneous exploration and locomotor activity, anxiety-related behavior, working and spatial memory, neuromuscular strength, sensorimotor processing, fine motor skills, emotional memory (Figure 2). At least two days interval between tests was ensured to minimize stress. All tests were conducted between h 10:00 am and 5:00 pm, and mice were habituated to the testing environment for at least 30 min before each session.

**Figure 7.**
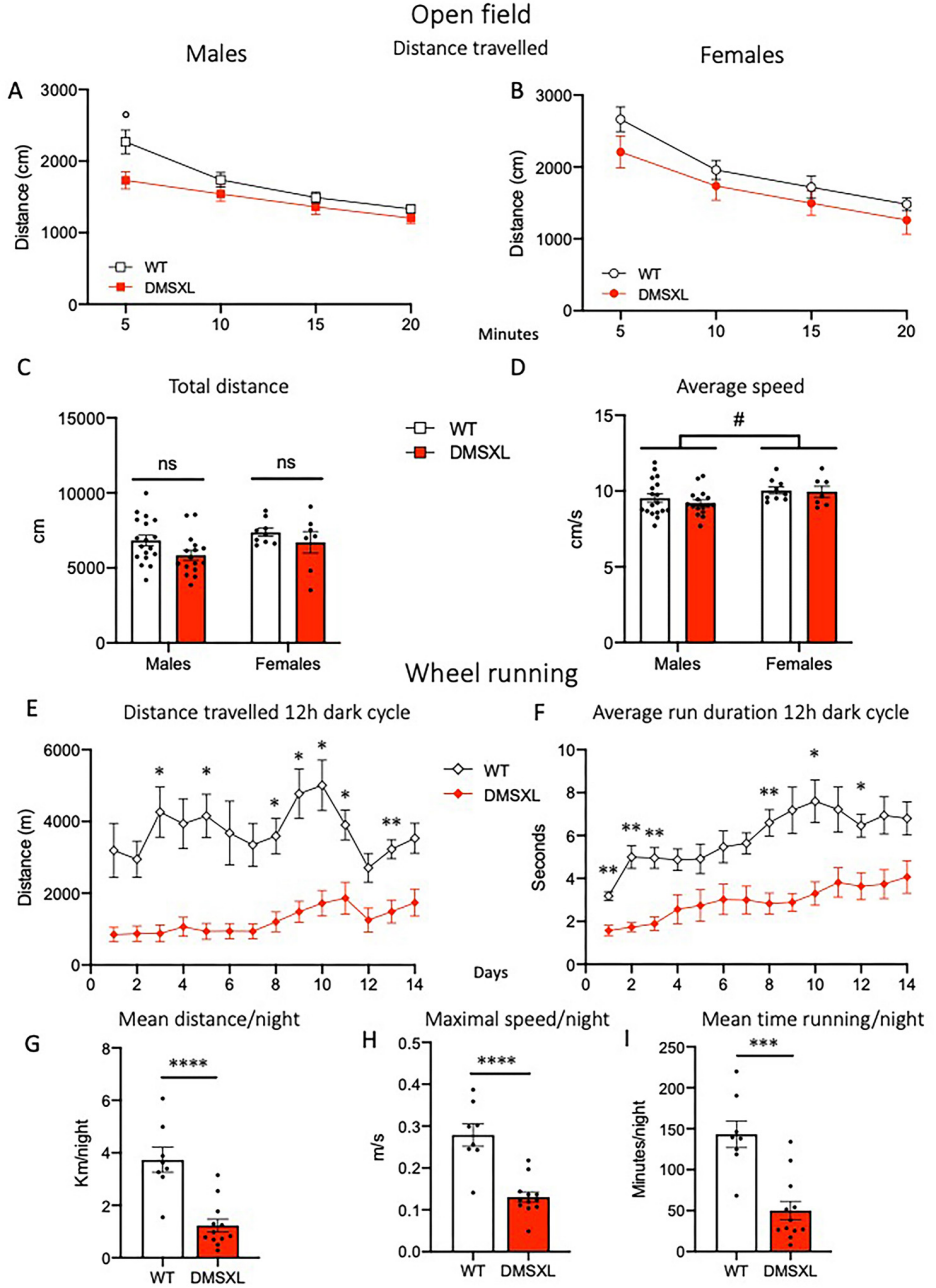
DMSXL mice show unaltered exploratory activity in the open field but reduced voluntary wheel running. Mice were tested in the open field at the age of 12 weeks (A-D). (A,B) Distance travelled in 5-min time bins in males (A) and females (B) and total distance (C) during the 20-min open field test. No main effect of Genotype was detected in either sex in the distance travelled (2-way RM-ANOVA; in males, °p = 0.05 Sidak's multiple comparisons); main effect of Time (F_(2,60)_ = 37.07 p < 0.0001 in males, F_(2,30)_ = 63.75 p < 0.0001 in females) revealed habituation in both sexes. A significant Time × Genotype interaction was found in males only (F_(3,96)_ = 3.5 p = 0.0178). (D) Female mice showed a higher average speed compared to males (2-way ANOVA effect of Sex F_(1,46)_ = 4.2, #p = 0.04).  Data are expressed as mean ± SEM. WT: males n = 18, females n = 10; DMSXL: males n = 16, females n = 7. One WT female was excluded in C and D as outlier. At the age of 16 weeks a subgroup of mice (WT n = 4/sex and DMSXL n = 6/sex) were singly housed with freely accessible running wheels and monitored 24/7 for 14 days (E-I). (E,F) Distance travelled (E) and average run duration (F) during 12-h dark cycle. DMSXL mice showed a reduced performance in both parameters. (E) 2-way RM-ANOVA effects of Genotype F_(1,18)_ = 26.06 p < 0.0001, Time F_(4,66)_ = 5.205 p = 0.0014, Time × Genotype F_(13,234)_ = 2.95 p = 0.0005. (F) Genotype F_(1,18)_ = 18.08 p = 0.0005, Time F_(4,64)_ = 9.63 p < 0.0001. Data are expressed as mean ± SEM. *p < 0.05, **p < 0.01 Sidak's multiple comparisons test. (G-I) During the 12-h dark phase, DMSXL mice ran significantly shorter distances (G), at a lower maximal speed (H), and for a reduced time (I) compared to WT littermates. Data are expressed as mean ± SEM. ***p < 0.001; ****p < 0.0001, unpaired t-test.

**Figure 8.**
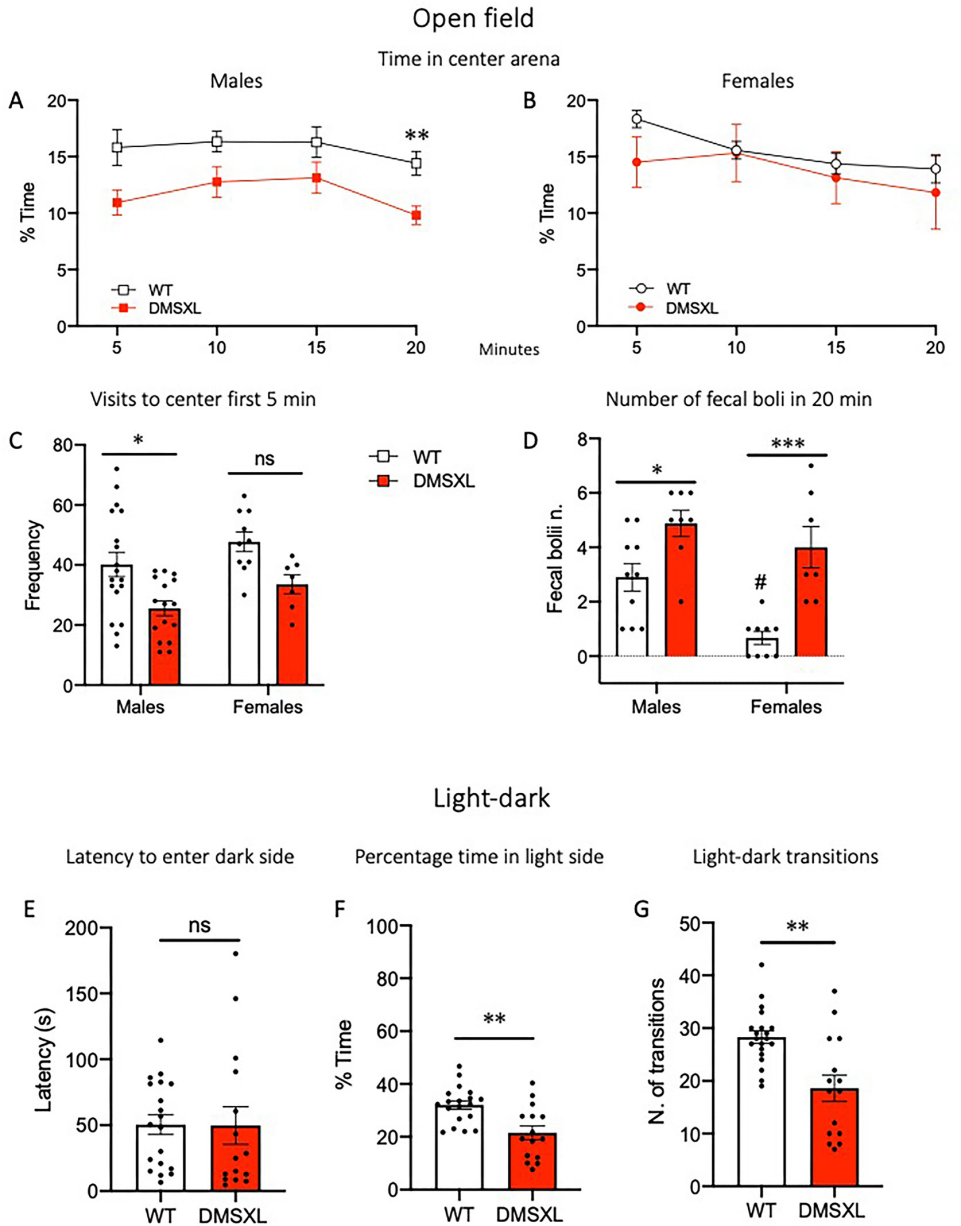
DMSXL mice show anxiety-like phenotype. Mice were tested in the open field at the age of 12 weeks for 20 min (A-D). (A) Male DMSXL mice spent less time in percentage in the center of the arena, RM-ANOVA effect of Genotype F_(1,32)_ = 7.51 p = 0.0099, **p = 0.0067 Sidak's multiple comparisons test, and showed a lower number of visits to the center of the arena in the first 5 min (C), 2-way ANOVA main effect of Genotype F_(1,47)_ = 13.46 p = 0.0006, *p = 0.01 Tukey's multiple comparisons test. In females, no genotype difference was observed (B,C). (D) Both male and female DMSXL mice and WT males showed greater number of fecal boli at the end of the test compared to WT littermates and to WT females respectively, 2-way ANOVA Genotype effect, F_(1,30)_ = 27.67 p < 0.0001, Sex effect, F_(1,30)_ = 9.49 p = 0.0044; *p = 0.036 WT *vs* DMSXL in males, ***p = 0.0005 WT *vs* DMSXL in females, #p = 0.011 in WT group, Tukey's multiple comparisons test. (E-G) A week later, mice performed the Light/Dark (L/D) test for 5 min, starting from the light side. While no difference between genotypes was observed in the latency to enter the dark side for the first time (E), DMSXL mice spent less time in percentage in the light side (**p = 0.0011 unpaired t-test) (F), and made fewer transitions (**p = 0.0021 Welch's t-test) (G), compared to WT. Data are expressed as mean ± SEM. In L/D test, since no main effect of sex or interaction was found in the two-way ANOVA, data from males and females were pooled (WT n = 28, DMSXL n = 23).

**Figure 9.**
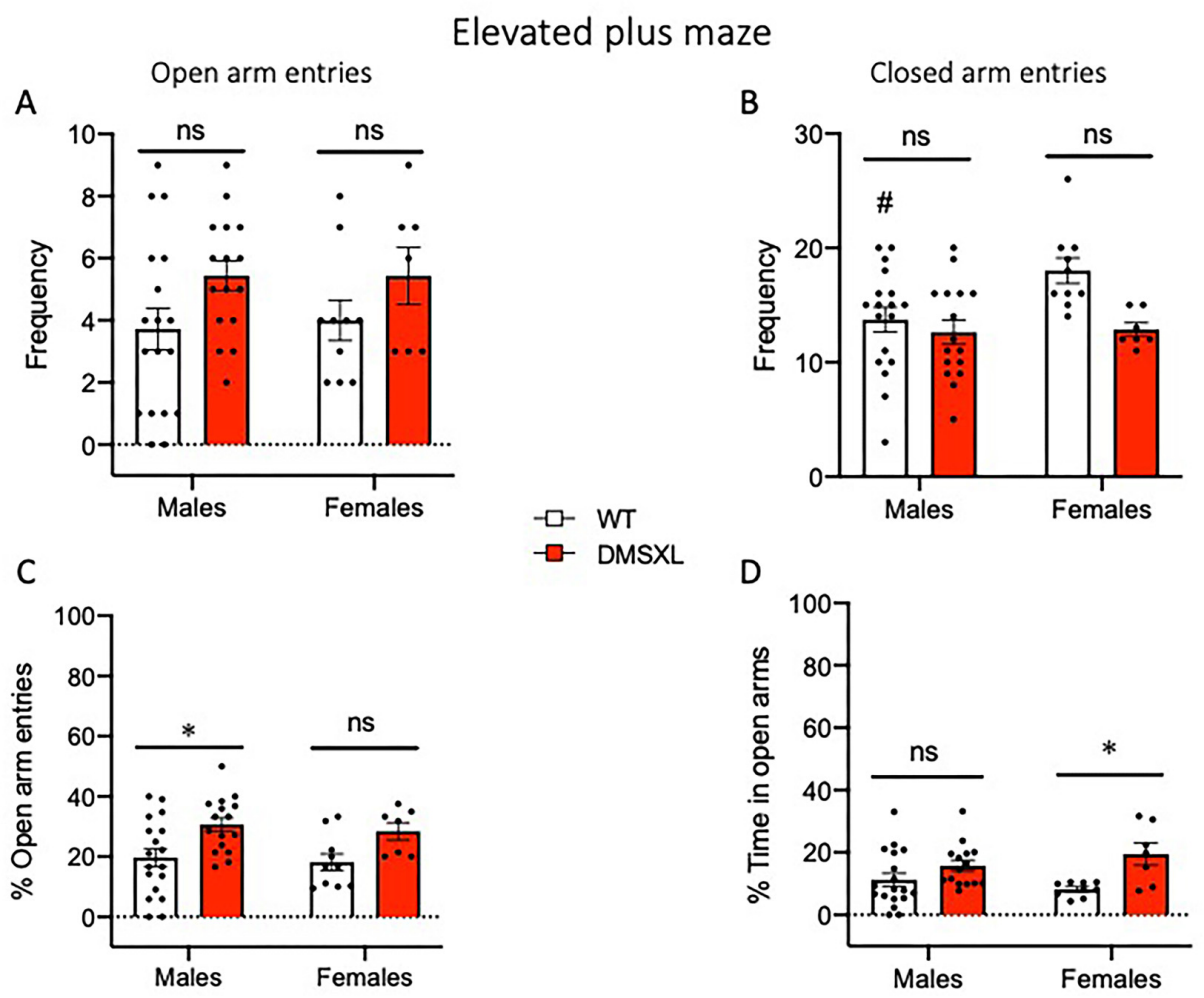
DMSXL mice show abnormal behavior in the elevated plus maze. Mice were tested in the elevated plus maze at 13 weeks of age for 5 min. (A,B) Both male and female DMSXL mice showed more entries in the open arms (A) and fewer entries in the closed arms of the apparatus compared to WT littermates (B). (A) 2-way ANOVA main effect of Genotype F_(1,47)_ = 4.85 p = 0.0325. (B) 2-way ANOVA main effect of Genotype F_(1,47)_ = 6.74 p = 0.0125, #p = 0.044 males *vs* females in WT group, Tukey's multiple comparisons test. (C,D) DMSXL mice exhibited a significantly higher percentage of entries into the open arms over the total entries (C) and spent significantly more time in the open arms (D) compared to WT littermates. (C) 2-way ANOVA main effect of Genotype F_(1,47)_ = 11.57 p = 0.0014, *p = 0.0184 WT *vs* DMSXL in males, Tukey's multiple comparisons test. (D) 2-way ANOVA main effect of Genotype F_(1,45)_ = 11.45 p = 0.0015, *p = 0.0257 WT *vs* DMSXL in females, Tukey's multiple comparisons test. No significant main effect of Sex or Sex × Genotype interaction was found for any of the parameters. Data are expressed as mean ± SEM. WT: males n = 18, females n = 10; DMSXL: males n = 16, females n = 7. In D, one WT female and one DMSXL male were excluded as outliers.

**Figure 10.**
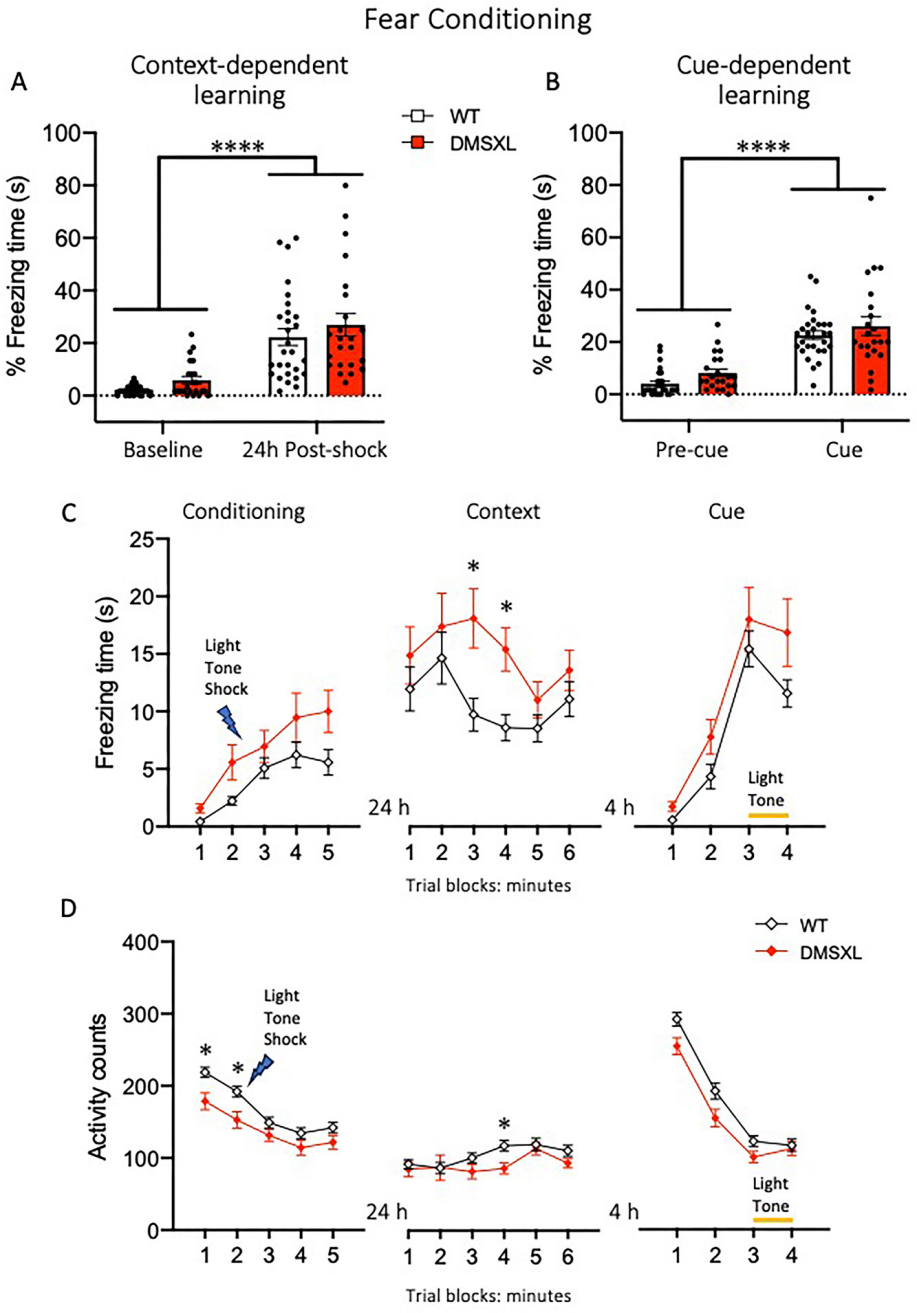
DMSXL mice show intact associative learning but altered freezing response in contextual fear conditioning. Mice were tested in the fear conditioning apparatus at the age of 16 weeks. (A,B) In both context- and cue-dependent learning paradigms, both WT and DMSXL mice showed increased percentage freezing time to the conditioned context after 24 h (A), or, 4 h later in a different context, to the light and tone stimuli previously associated to a foot-shock (B). (A) 2-way RM ANOVA context learning F_(1,49)_ = 62.58 p< 0.0001; (B) cue learning F_(1,48)_ = 117.7 p< 0.0001. During the conditioning session, DMSXL mice showed a mild but significant increase in freezing response and a corresponding decrease in activity across time compared to WT littermates, in particular during the first two minutes (C,D left panels). (C) 2-way RM ANOVA Genotype effect, F_(1,49)_ = 6.83 p = 0.0118, Time effect F_(3155)_ = 15.83 p < 0.0001; (D) 2-way RM ANOVA Genotype effect, F_(1,49)_ = 8.46 p = 0.0054, Time effect F_(3165)_ = 44.77 p < 0.0001, *p < 0.05 WT *vs* DMSXL Sidak's multiple comparisons test. During the context recall session, DMSXL mice showed a significantly higher freezing response over time compared to WT (C, middle panel) 2-way RM ANOVA Genotype effect, F_(1,49)_ = 5.0 p = 0.029, Time effect F_(3155)_ = 15.83 p = 0.006, *p < 0.05 WT *vs* DMSXL Sidak's multiple comparisons test, which was not accompanied by a general reduction in locomotor activity (D, middle panel) 2-way RM ANOVA Genotype effect not significant, Time effect F_(4177)_ = 4.59 p = 0.0022, *p < 0.05 WT *vs* DMSXL Sidak's multiple comparison test. During the cue session, both WT and DMSXL mice showed increased freezing response and decreased activity when exposed to light and tone stimuli (C,D right panels) 2-way RM ANOVA Time effect F_(2107)_ = 53.55 p < 0.0001 (C), F_(3135)_ = 203 p < 0.0001 (D), with DMSXL mice displaying reduced activity counts compared to WT (D, right panel) 2-way RM ANOVA Genotype effect, F_(1,49)_ = 5.5 p = 0.023. Data are presented as mean ± SEM, data from males and females were pooled (WT n = 28, DMSXL n = 23). In (B) one DMSXL mouse was excluded as outlier.

**Figure 11.**
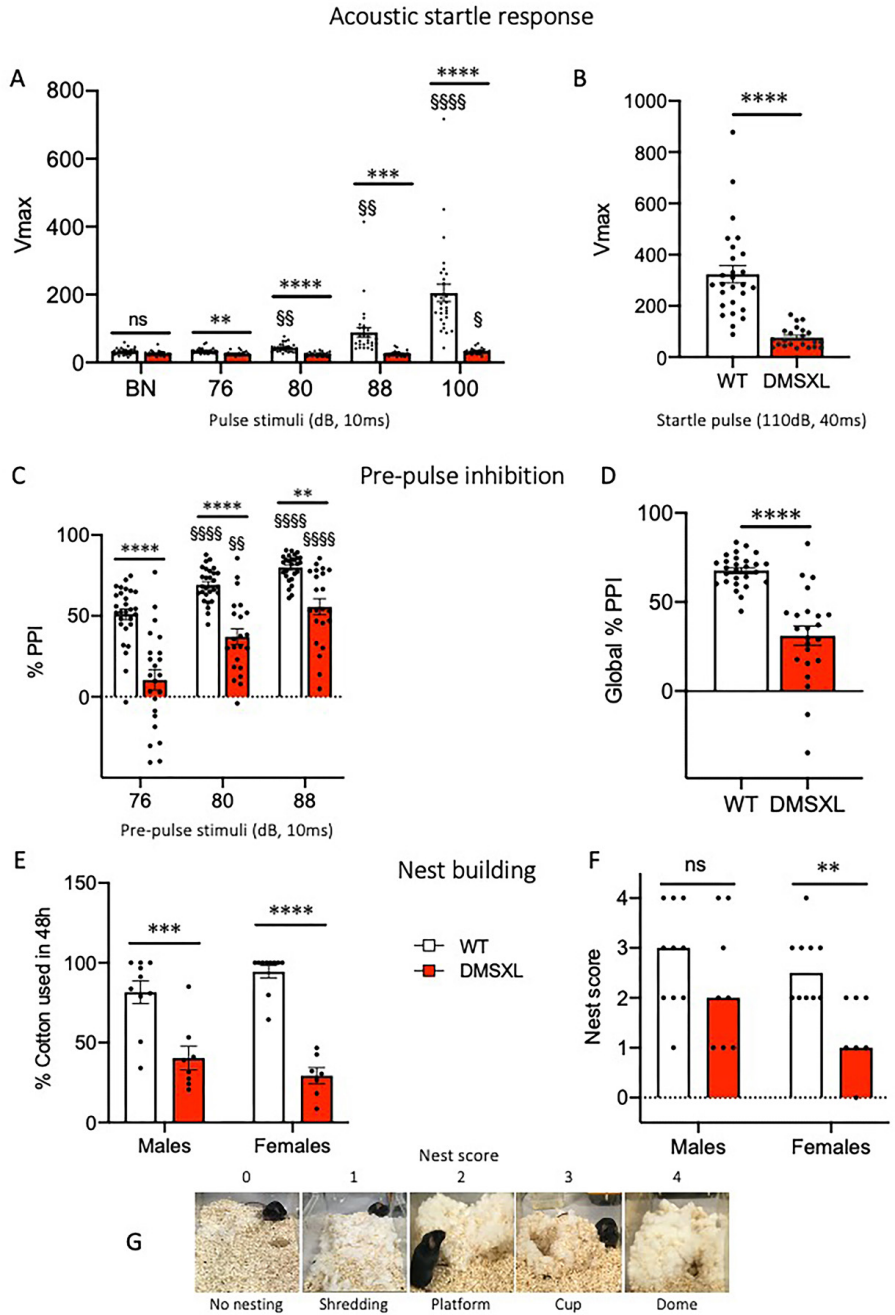
DMSXL mice show deficits in sensorimotor gating and fine motor skills. At the age of 15 weeks, mice were tested for sensorimotor gating in the Acoustic startle response/Pre-Pulse Inhibition (PPI) test (A-D) and for their ability to build their nest (E-G). (A) In the Acoustic startle/PPI test, mice received a random sequence of pulse-alone trials (76–100 dB, 10 ms) or pre-pulse (10 ms) + startle pulse (110 dB, 40 ms) trials. DMSXL mice showed a clear deficit in the peak response (Vmax) to all the acoustic stimuli compared to WT, responding to the 100 dB pulse only, 2-way RM-ANOVA effects of Genotype F_(1,49)_ = 56.64, Stimulus F_(2,77)_ = 28.82, and Genotype×Stimulus F_(4196)_ = 24.63, all p < 0.0001; **p < 0.01, ***p < 0.001, ****p < 0.0001 WT *vs.* DMSXL; § p < 0.05, §§ p < 0.01, §§§§ p < 0.0001 *vs* BN in genotype groups, Bonferroni's multiple comparisons. (B) DMSXL mice showed a reduced response to the 110 dB startle pulse: ****p < 0.0001 WT *vs.* DMSXL Welch's t-test. Nonetheless, inhibition of the response, reported as percentage, occurred in DMSXL mice when the startle pulse was preceded by a pre-pulse, although to a significantly lesser extent than in WT mice. (C) 2-way RM-ANOVA effects of Genotype F_(1,49)_ = 42.45, Stimulus F_(2,98)_ = 50.89, p < 0.0001; **p < 0.01, ****p < 0.0001 WT *vs.* DMSXL; §§ p < 0.01, §§§§ p < 0.0001 *vs* 76 in genotype groups, Bonferroni's multiple comparisons. (D) Global %PPI: ****p < 0.0001 WT *vs.* DMSXL Welch's t-test. Since there were no sex differences, the data of male and female individuals were pooled together (WT n = 28, DMSXL n = 23). (E-G) To evaluate fine motor skills, mice were singly housed and provided with cotton to be used for nest building. (E,F) Both male and female DMSXL mice showed reduced nest building ability compared to WT littermates, measured as the percentage of cotton used after 48 h, and a lower nest score. (E) 2-way ANOVA Genotype effect, F_(1,31)_ = 74.55 p < 0.0001; ***p < 0.001, ****p < 0.0001 WT *vs.* DMSXL in males and females respectively, Sidak's multiple comparisons test. (F) The Kruskal Wallis test revealed a significant difference across the four groups (p = 0.0267). Pairwise comparisons using Mann-Whitney U tests showed a significant reduction in nest-building scores in DMSXL females compared to WT (**p = 0.0043, U = 7.5). (G) Representative images of nest shape and the relative score. Data are expressed as mean ± SEM or median. WT: males n = 10, females n = 10; DMSXL: males n = 8, females n = 7.

**Figure 12.**
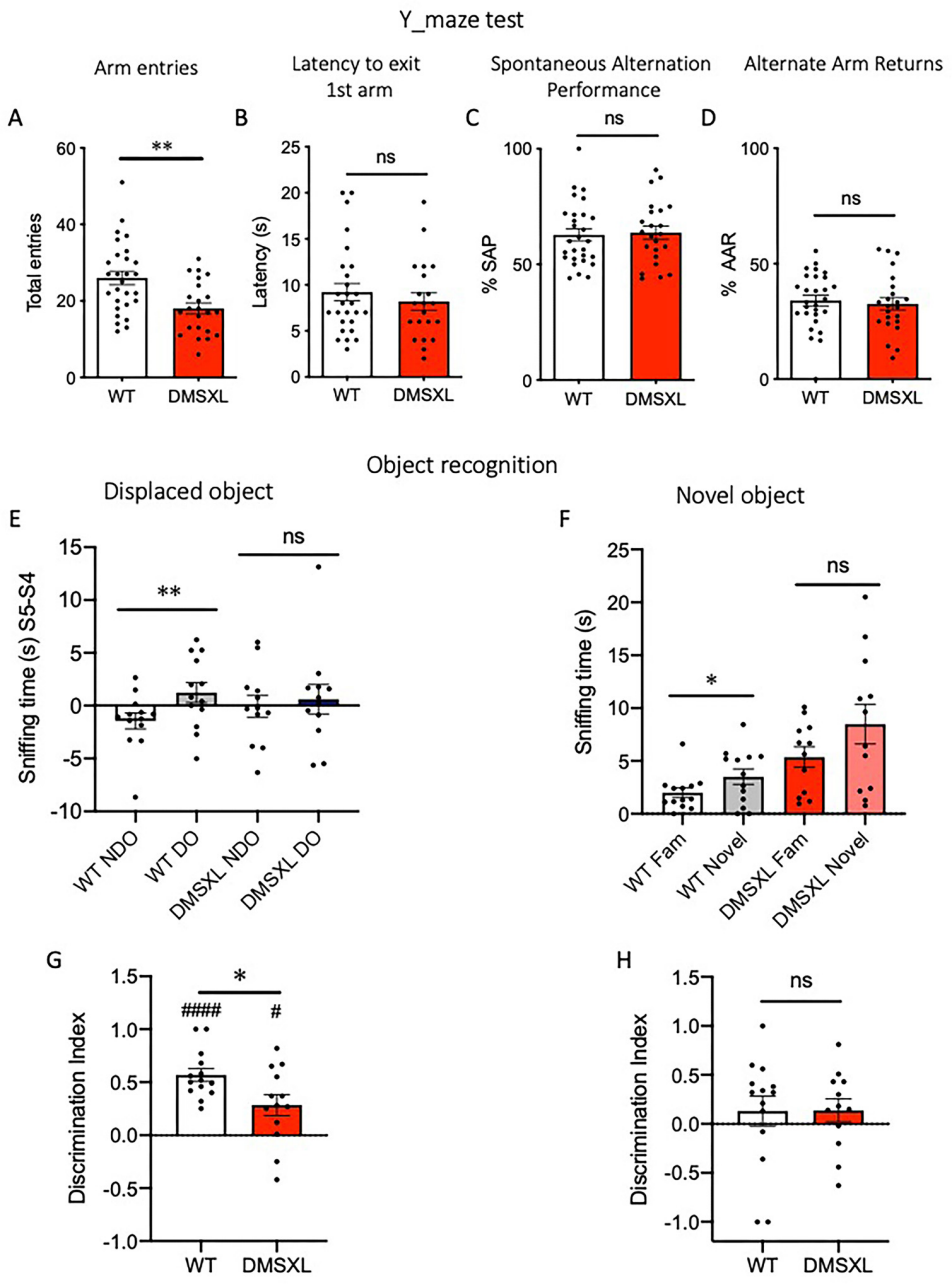
DMSXL mice show intact working memory but display deficits in object recognition. Working memory was evaluated using the Y-maze spontaneous alternation task, at the age of 14 weeks (A-D). (A) DMSXL mice showed a significant reduction in total arm entries compared to WT (unpaired t-test, t = 3.4, **p = 0.0013). No differences were observed in latency to exit the first arm (B), spontaneous alternation performance (%SAP) (C), or alternate arm returns (%AAR) (D). Data are expressed as mean ± SEM. Data from male and female mice are pooled; WT n = 28, DMSXL n = 23. At the age of 20 weeks a subgroup of mice performed the displaced/novel object recognition task (E-H). After three habituation sessions (S2-S3-S4) with two familiar objects, mice had to explore the same objects, one of which was relocated in a different position in the arena (S5). (E) WT mice spent significantly more time investigating the displaced object (DO) compared to the non-displaced one (NDO) **p = 0.0071, t = 3.24 paired t-test, whereas this difference was not observed in DMSXL mice. (F) In the novel object session (S6), WT mice also showed a significantly higher exploration of the novel object compared to the familiar one (*p = 0.024, t = 2.57 paired t-test), a difference not significant in DMSXL mice. Discrimination index for the displaced object (G) and novel object (H) in WT and DMSXL mice, calculated as the sniffing time difference of displaced/novel object with the familiar one, over total exploration time. (*p = 0.02, t = 2.483 unpaired t-test; #### p < 0.0001; #p = 0.014, One sample t-test vs. 0). Data are presented as mean ± SEM, data from males and females were pooled (WT n = 13–14, DMSXL n = 12–13).

#### Body weight and grip strength test

Male and female WT and DMSXL mice were weighed weekly throughout the behavioral testing period (age 12–20 weeks). From the age of 17 weeks, only animals of the second cohort were weighed, and, due to other scheduled procedures, only 6 WT males and 8 WT females were weighed at age 20 weeks (Table S2).

Grip strength test was performed as described^74^ at age 14 weeks. Briefly, mice were gently pulled by their tail while gripping with the all four paws to a small grid attached to a dynamometer (Bioseb, France). Peak force in grams was recorded over three consecutive trials with one minute of inter-trial interval. Mean values were considered for the analysis.

#### Open field test

The open field test was used to assess spontaneous exploratory activity and anxiety-like behavior, which are inferred from the natural conflict between the drive to explore and the aversion to open spaces. Mice were individually placed in a square arena (cm 44 × 44 × h 33.5, made of gray PVC) and allowed to explore freely for 20 min. The apparatus was located in a dimly lit (approximately 100 lux inside the arena), quiet testing room. Locomotor activity was recorded in 5-min intervals for a total of 20 min by an automated video tracking system (ViewPoint, Lyon, France) and expressed as distance traveled in cm. Anxiety-like behavior was evaluated based on the number of visits to the center of the arena during the first 5-min interval, and the percentage of time spent in the center area over each 5-min interval across the 20-min session.^75^ Immediately after the session, the number of fecal boli left in the arena was counted as an additional index of anxiety-related emotionality.^76^

#### Wheel running activity

A subgroup of 20 male and female WT and DMSXL mice were singly housed into Type II long home cages (Ebeco, Germany), each equipped with a running wheel (TSE Systems, Bad Homburg, Germany). Food and water were available *ad libitum*, and nest paper was supplied in each cage. For the DMSXL group, some food pellets were also placed directly inside the cage to facilitate access. Mice were monitored continuously for running wheel activity over a two-week period using the PhenoMaster software (TSE Systems, Germany).

The following parameters were analyzed for the 12-h dark phase, as previously described^77^ : (1) distance travelled (meters) and mean distance run over the two weeks (Km); (2) average duration (seconds) of running episodes in which the velocity exceeded 30 rpm; (3) maximal speed (m/s), calculated from daily values as the mean of peak speed values sampled every 5 min; (4) mean running time over the two weeks (minutes). To monitor health status, mice were checked daily and weighed every 3–4 days.

#### Light/dark test

This test was used to evaluate anxiety-related phenotypes. It is based on the innate aversion of mice to brightly lit open spaces and the tendency to explore a novel environment.^78^ The apparatus consisted of a standard rat cage (cm 36.5 × 20.7×h14) equally divided into a light (cm 18 × 20.7, 520 lux) and a dark compartment of the same size, connected by a small opening.^79^ Each mouse was gently placed in the illuminated side, facing the wall opposite to the dark compartment. The latency to enter the dark compartment (all four paws inside) was recorded manually as the primary measure. After the first entry, the number of transitions between the light and dark compartments and the percentage of time spent in the illuminated side were measured for a duration of 5 min, as indicators of exploratory activity and anxiety-like behavior, respectively.

#### Elevated plus maze test

The elevated plus maze (EPM) was used to evaluate anxiety-related behaviors in mice, as originally described by Lister.^80^ The apparatus was made of dark grey PVC and consisted of two opposing open arms (30 × 5 cm, with 0.3 cm edge barriers), two opposing closed arms (30 × 5 cm, with 13 cm high walls), and a central square platform (5 × 5 cm) connecting the arms. The maze was elevated 50 cm above the floor. Lighting levels were set at approximately 80 lux in the open arms and 30 lux in the closed arms. Each mouse was placed on the central platform facing one of the open arms, and behavior was recorded for a 5-min test session. Video recordings were later analyzed manually using a custom-made software (Mouse Watch 1.1, R. H. Butler, personal communication). The following parameters were measured: number of entries into open and closed arms (defined as all four paws entering the arm), and the time spent in each arm type.^79^

#### Fear conditioning test

Fear learning and memory were assessed using an automated conditioning system (Freeze Monitor, San Diego Instruments, CA, USA), following previously described procedures.^79,81^ The protocol consisted of three phases:

Conditioning session (day 1): each mouse was placed in the test chamber (25 × 25×h19 cm, 65 lux) and allowed to explore it for 2 min. A conditioned stimulus (CS), consisting of a tone and light combination (92 dB, 140 lux, 20 s duration), was then presented and paired during the final second with a mild foot shock (unconditioned stimulus, US, 0.4 mA, 1 s). After the CS–US pairing, the mouse remained in the chamber for a total session duration of 5 min.

Context session (day 2, 24 h later): The mouse was returned to the same testing chamber for 6 min, with no CS or US presentation. Freezing behavior was recorded as an index of contextual memory.

Cue session (day 2, 4 h after the context session): The mouse was placed in a modified chamber to provide a novel context: the apparatus was rotated 90°, covered with black PVC plates on floor and walls, room light was turned off (0 lux), and a cinnamon scent was introduced in the chamber. The mouse explored for 2 min before being re-exposed to the CS (tone + light, 2 min). In all phases, freezing behavior, defined as the absence of any movement except for respiration, and activity were automatically recorded. Freezing was considered to occur when the system detected fewer than three infrared beam breaks within a 5-s period, with a minimum freezing episode lasting 2 s.^82^ Freezing data were expressed as the percentage of time spent freezing in one or two-minute trial blocks.

#### Acoustic startle reflex and pre-pulse inhibition

The acoustic startle reflex (ASR) is a rapid motor response to a sudden auditory stimulus, typically characterized by whole-body flinching. This response can be modulated when a weaker auditory stimulus (pre-pulse) precedes the startling pulse, a phenomenon known as pre-pulse inhibition (PPI), which provides a functional measure of sensorimotor gating, the brain's ability to filter and process sensory information.^83^ ASR and PPI were assessed using the SR-Lab apparatus (San Diego Instruments, San Diego, CA, USA). Mice were individually placed in the chamber, and after a 5-min acclimation period with 72 dB background noise (BN), they were exposed to a randomized series of 9 trial types: startle pulse alone (white noise, 110 dB 40 ms); four pulse-alone trials (76, 80, 88, or 100 dB, 10 ms); three pre-pulse + startle pulse trials, in which a pre-pulse of 76, 80, or 88 dB (10 ms) preceded the startle pulse (110 dB, 40 ms) by 50 ms; one background noise trial (72 dB, BN) to assess baseline activity. Each trial type was presented 10 times in pseudorandom order, with an inter-trial interval (ITI) varying between 20 and 30 s (average 25 s). The startle response was recorded for 65 ms after the onset of the startle pulse, and the peak startle amplitude (Vmax) was used for analysis. PPI was calculated using the formula: % PPI=100×(Vmax startle trial -Vmax pre-pulse + startle pulse trials) / Vmax startle trial.^74^

#### Nest building ability

To evaluate fine motor skills, we used the non-invasive nest building.^84^

Mice were single housed in standard cages containing only bedding material, without nesting paper. Food pellets were placed directly in the cage, and wet chow was provided to DMSXL mice. A pre-weighed amount of cotton (8–9 grams) was placed on the food grid, requiring the mice to use their forepaws to pull it through the bars and construct a nest. Cotton use and nest building were evaluated after 24 and 48 h. At each time point, the remaining cotton on the grid was weighed, and the nest was scored based on its shape and structure using a standardized 5-point scale (0 = no nesting, 4 = a well-structured dome-shaped nest, Figure 11G). The data reported refer to the 48-h time point.

#### Y-maze test

The Y-maze is used to assess spontaneous alternation which is considered as an index of active retrograde working memory and is driven by the innate curiosity of rodents to explore previously unvisited areas. The apparatus, made of opaque grey PVC, consisted of three identical arms (cm 30 × 9×h 15) arranged at 120° angles to form a Y-shape, and connected by a central equilateral triangular platform. Mice were individually placed at the end of one arm, facing the wall, and allowed to freely explore the maze for 5 min under moderate lighting conditions (approximately 100 lux in the central area). The starting arm was alternated between animals to avoid positional bias. An arm entry was recorded when all four paws entered the arm. A spontaneous alternation was defined as successive entries into each of the three arms on overlapping triplet sets (e.g., ABC, BCA, CAB, etc.). The percentage of spontaneous alternation performance (% SAP) was calculated as: % SAP = (Number of actual alternations / [Total number of arm entries - 2]) × 100. Additionally, alternate arm returns (AAR) were scored (e.g., ABA, BCB, CAC, etc.). The total number of entries was used as a measure of locomotor activity, while the latency to exit the starting arm was used as an indicator of emotionality or hesitation behavior.^74^

#### Open field with objects

The task, simplified and adapted from Mandillo et al.^85^ and Branchi & Ricceri, et al.,^86^ was used to assess both spatial and recognition memory in the open field arena. The test was conducted over six consecutive sessions, each lasting 5 min and separated by 5-min intervals, during which mice were placed back to home cage. In session 1, mice were placed in the empty open field arena for 5 min. In sessions 2–4, two distinct objects (familiar objects) were placed symmetrically ∼8 cm from one of the arena walls, on which a striped black-and-white paper was fixed to aid spatial orientation (Supplementary Figure S4). In session 5, one of the two familiar objects (the same across all animals) was relocated to a different position (displaced object). In session 6, the displaced object was replaced by a novel object. Object exploration was defined as direct sniffing (nose within ∼2 cm from the object) or approaching, i.e., the animal's head and body oriented toward the object with the nose clearly directed at it, typically involving a forward stretching posture. Sessions were video-recorded (four mice simultaneously), and the videos were subsequently analyzed manually. The following measures were calculated: Preference for the displaced object – difference in exploration time between the displaced object in session 5 vs. the same object in session 4; Novel object recognition – comparison of time spent exploring the novel vs. familiar object in session 6; Discrimination index – calculated as (time exploring displaced/novel object – time exploring familiar object) / total exploration time, in session 5 or 6, respectively.

#### Statistical analysis

Statistical analyses were performed using GraphPad Prism version 8.3.1 (GraphPad Software, San Diego, CA, USA) or StatView version 5.0.1 (SAS Institute Inc., Cary, NC, USA). Data were tested for normality using the Shapiro-Wilk and Kolmogorov-Smirnov tests. Continuous variables were analyzed with unpaired t-test or Welch's corrected t-tests, paired t-tests, one-way or two-way ANOVA (with repeated measures where applicable), and non-parametric tests such as Kruskal–Wallis or Mann–Whitney U tests. When multiple comparisons were made, appropriate corrections (Sidak's, Tukey's, or Bonferroni's post-hocs) were applied. Sex and genotype were considered as independent factors where appropriate. When no main effect of sex or sex × genotype interaction was detected, data from males and females were pooled to increase statistical power. For each parameter outliers were identified using Grubbs’ method (Alpha=0.05) and excluded when justified. Data are presented as mean ± SEM or median, as appropriate, and significance was set at p < 0.05.

## Results

### Longitudinal analysis of exploratory and anxiety-like behaviors in DMSXL mice from French cohorts

An overview of the experimental timeline and the distribution of behavioral tests across the different mouse cohorts studied is provided in Figure 1. To assess general locomotor activity, exploratory behavior, and anxiety-like responses over time, we performed the open field test on a first cohort of male and female WT and DMSXL homozygous mice (n = 16–17 per group) at 1, 2, 4, 8, and 12 months of age, during 30 min sessions. Behavioral parameters measured included total distance traveled, as well as the percentage of time spent and distance traveled in the central versus peripheral zones of the arena, which together reflect locomotor performance and anxiety-related behavior (Figure 1).

Over the course of aging, DMSXL mice exhibited significant differences in body weight and open field behavior compared to WT littermates (Figure 3). As expected, both male and female DMSXL mice had significantly lower body weight than WT controls at all time points assessed (Figure 3A) as previously described.^54^ In the open field test, an age-dependent effect was observed for total distance traveled over the 30-min session in both sexes, with a general decline in activity over time (Figure 3B). No significant genotype differences were detected; however, at 8 and 12 months of age, DMSXL males tended to remain slightly more active than their WT counterparts. This suggests that neither the smaller body size of DMSXL mice nor their potential muscle weakness significantly affects their spontaneous locomotor activity in the open field. Time spent and distance traveled in the center of the arena, two commonly used indices of anxiety-like behavior, both showed a significant overall genotype effect, with DMSXL males exhibiting higher values compared to WT (Figure 3C and D). These differences were not observed at 1 month of age. This pattern suggests a genotype-dependent reduction in anxiety-like behavior or altered risk assessment in DMSXL mice, emerging at 2 months and persisting with age. No significant genotype differences were detected in females for these parameters, although a difference was noted at 12 months; this finding should be interpreted with caution due to the smaller sample size at this time point (n = 6 per group). To better understand why DMSXL mice, particularly males, spent more time in the center of the arena, we further analyzed the total number of entries into the central zone (Supplementary Figure S5). A statistically significant effect of age and genotype was observed only in males, with DMSXL mice showing a progressive increase in center entries relative to WT, reaching significance from 8 months of age. These findings confirm that the increased time spent in the center is not attributable to reduced mobility or passive behavior, but rather reflects a genuine behavioral phenotype, possibly related to altered anxiety processing or risk evaluation in DMSXL mice.

To assess novelty-induced inhibition behavior, we analyzed the total distance traveled and the number of entries into the center during the first 5 min of the open field test at different ages (Figure 4A). A significant age effect was observed for total distance in both sexes with a general decline over time. A genotype effect was detected only in females, with DMSXL mice showing reduced activity compared to WT at 1 month of age. For the number of center entries, no significant main effects of age or genotype were detected in females. However, post hoc analyses revealed a significant reduction in center entries in 1-month-old DMSXL females compared to WT, consistent with their lower overall activity and suggesting novelty-induced inhibition at this early stage. This phenotype appeared transient, as it was no longer detectable after 2 months of age. In contrast, DMSXL males exhibited an opposite pattern later in life, showing an increased number of center entries compared to WT at 8 months of age. Together, these results indicate an early and transient inhibition of exploratory behavior in DMSXL females, whereas males display a late-onset increase in center exploration.

Given the alterations observed in the open field test, we next sought to determine whether these behavioral differences extended to another widely used paradigm of anxiety-like behavior. We therefore employed the elevated plus maze (EPM), a well-established assay that probes risk assessment and exploratory drive in a novel anxiogenic environment. Based on our previous findings showing more pronounced behavioral alterations in male DMSXL mice, we focused this analysis on males from a second cohort and performed the EPM test at 1, 2, and 4 months of age to capture potential age-dependent effects. In the elevated plus maze test, significant age and genotype effects was observed for total distance traveled (Figure 4B). At 1 month of age, DMSXL male mice exhibited a marked reduction in locomotor activity compared to their WT littermates associated with significantly reduced entries into the closed arms reflecting early hypoactivity (Figure 4B). This difference progressively decreased with age, and by 4 months, total distance traveled was comparable between genotypes. When evaluating anxiety-like behavior through the percentage of time spent in the open arms, we found a significant age-dependent decrease across groups, indicating increased avoidance behavior with age. No difference was detected between genotypes at 1 or 2 months. However, at 4 months, DMSXL mice spent a significantly greater proportion of time in the open arms than WT mice, suggesting altered risk assessment and impaired inhibitory control. A similar age-related decline was observed in the distance traveled within the open arms. While no significant genotype effect was observed at earlier ages, DMSXL mice exhibited significantly greater open-arm exploration at 4 months compared to WT controls, as evidenced by an increased number of entries into the open arms at that age (Figure 4B). Together, these results suggest that DMSXL mice exhibit early hypoactivity and later-emerging alterations, which may reflect age-dependent changes in emotional reactivity or decision-making processes.

These findings are consistent with the behavioral profile observed in the open field test, where DMSXL males from 2 months displayed greater exploratory behavior in the anxiogenic center zone, reflected by increased percentage of time, number of entries, and distance traveled in the center. Together, these data indicate that DMSXL mice exhibit early hypoactivity, possibly related to novelty-induced inhibition at one month, followed by the emergence of atypical anxiety-like behavior in adulthood, possibly reflecting dysregulated emotional reactivity or reduced avoidance responses to anxiogenic situations.

### Cognitive function in DMSXL mice: working and recognition memory performance

To investigate potential cognitive impairments in DMSXL mice, we assessed two complementary forms of memory using the spontaneous alternation Y-maze and the novel object recognition tests. These paradigms probe distinct cognitive domains and operate on different temporal scales. The Y-maze test evaluates spatial working memory, based on the natural tendency of rodents to alternate between arms during free exploration. This task requires the ability to retain and update spatial information over short time intervals and is sensitive to deficits in executive function and attention. In contrast, the novel object recognition test probes recognition memory**,** as it relies on the animal's ability to discriminate between familiar and novel objects. In our protocol, the recognition phase was performed 24 h after the familiarization session, thereby assessing memory consolidation over a prolonged delay.

For the Y-maze, the second cohort of male mice was tested at 1, 2, and 4 months of age (Figure 5A). At 1 month, DMSXL mice exhibited a significant reduction in total distance traveled compared to WT littermates as well as a decrease in the total number of arm entries indicating reduced exploratory activity. These differences were no longer observed at 2 and 4 months, where both parameters were comparable between genotypes. Spontaneous alternation performance did not differ between WT and DMSXL mice at any age tested. Both genotypes displayed similar alternation percentages across time points, suggesting preserved spatial working memory in DMSXL mice under these conditions. Finally, latency to the first arm entry did not differ between genotypes at any age, further supporting the absence of initiation deficits or reduced exploratory drive in this task.

The novel object recognition test was performed in two independent cohorts of male WT and DMSXL mice (n = 8 per group) at 2 and 4 months of age, using a 24-h delay between the familiarization and test phases. At 2 months, both WT and DMSXL mice spent significantly more time exploring the novel object compared to the familiar one (Figure 5B). These results indicate that recognition memory is preserved in DMSXL mice at this age. At 4 months, WT mice also showed a significant preference for the novel object. DMSXL mice tended to prefer the novel object as well, but this difference did not reach statistical significance, suggesting a less robust novelty discrimination. However, this difference was not captured by the discrimination index (Figure 5C), likely due to greater inter-individual variability and reduced total exploration time in some animals. Together, these findings suggest that long-term recognition memory is preserved in DMSXL mice at 2 months, but may become less consistent with age, as reflected by a weaker and more variable novelty preference at 4 months.

### Assessment of social interaction and novelty preference in the three-chamber test

To extend our behavioral characterization of DMSXL mice to the social domain, we next employed the three-chamber test, a three-chambered apparatus used to evaluate sociability and preference for social novelty in rodents. This test allows assessment of the animal's interest in a conspecific versus an inanimate object, as well as its ability to discriminate between a familiar and a novel social partner. Given that social impairments have been described in DM1 patients, we aimed to determine whether DMSXL mice display alterations in social interaction behaviors that may reflect key features of the CNS phenotype.

During the sociability phase, both WT and DMSXL mice spent significantly more time in the compartment containing the unfamiliar mouse (Stranger 1) compared to the empty one, indicating a preserved preference for social interaction in both genotypes, although the effect was markedly stronger in WT mice than in DMSXL (Figure 6A). In the social novelty phase, WT mice spent significantly more time in the chamber containing the novel stranger (Stranger 2) compared to the familiar one (Stranger 1), whereas DMSXL mice did not display a significant preference, indicating impaired social discrimination (Figure 6B). These findings were further supported by sniffing behavior analysis. During the sociability phase, WT mice showed a significantly higher number of sniffing bouts directed at Stranger 1 than at the empty cage, whereas this preference was not significant in DMSXL mice. However, in the social novelty phase, the number of sniffing bouts did not significantly differ between Stranger 1 and Stranger 2 in either genotype, suggesting that this parameter may be less sensitive for detecting social novelty preference under our experimental conditions.

Together, these results suggest that while WT mice exhibit intact sociability and social novelty recognition at 5 months, DMSXL mice show a reduced drive for social interaction and impaired social discrimination.

### Reevaluation of body weight and grip strength in DMSXL male and female mice

To complement the longitudinal behavioral profiling performed in Paris, further analyses were conducted at the CNR Institute of Biochemistry and Cell Biology (CNR-IBBC, Italy) on two independent cohorts of DMSXL and WT mice between the age of 12 and 20 weeks, corresponding to approximately 3 and 5 months of age. This complementary cross-sectional approach allowed a more detailed phenotypic assessment during a critical age window, using a comprehensive behavioral test battery (Figure 2).

Mice body weights were recorded weekly throughout the duration of the test battery. As already reported,^54,66,87^ homozygous DMSXL mice exhibited persistently lower body weights, with an approximate overall reduction of 40% in males and 36% in females compared to their wild type littermate controls (Supplementary Figure S3 A). To measure muscle strength, the grip test was performed at the age of 14 weeks. Both male and female DMSXL mice displayed reduced grip force assessed with the four paws, with a decrease of approximately 24% in males and 37% in females compared to wild-type controls. This phenotype, previously reported in adult females,^54^ was also confirmed here in adult males (Supplementary Figure S3 B).

### DMSXL mice show reduced locomotor activity and abnormal emotional responses

Spontaneous locomotor activity was assessed in two different conditions: for 20 min in the open field at the age of 12 weeks, and continuously over 14 days in the home cage by monitoring voluntary running wheel activity (age 16–18 weeks). No significant differences in distance travelled or habituation were found between genotypes in either sex when tested in the open field arena (Figure 7A to C); however, females, regardless of genotype, displayed a higher average speed compared to males (Figure 7D). Conversely, DMSXL mice showed significantly reduced activity compared to wild-type littermates during their active phase (12-h lights off), when monitored in the home cage using running wheels. All running wheel activity parameters (distance, duration of running episodes, maximal speed, running time) were markedly decreased in DMSXL mice (Figure 7E-I). Interestingly, this phenotype was consistent with the reduced spontaneous activity recorded 24/7 using the Digital Ventilated Cage (DVC^®^) system, as we previously reported.^59^

As for the evaluation of anxiety-related behaviors, when tested in the open field, male but not female DMSXL mice spent a significantly lower percentage of time in the center of the arena (Figure 8A and B), and, additionally, showed a reduced number of visits to the center in the first 5 min (Figure 8C). Both male and female DMSXL mice produced more fecal boli compared to wild-type at the end of the test. While we cannot entirely exclude gastrointestinal (GI) alterations in DMSXL mice (a significant feature in DM1 patients), we interpret increased number of fecal boli primarily as an indicator of heightened anxiety (Figure 8D). At the age of 13 weeks mice were further tested in the light/dark and elevated plus maze tasks. In the light/dark test, a significantly reduced time spent in the lit compartment was observed in DMSXL mice (Figure 8F), thus indicating increased anxiety-like behavior. No difference was observed in the latency to enter the dark side for the first time between wild-type and DMSXL mice; however, DMSXL mice made fewer light-dark transitions (Figure 8E and G).

Interestingly, in the elevated plus maze test we observed an opposite trend. Specifically, DMSXL mice, regardless of sex, exhibited an increased exploration of the open arms compared to wild-type littermates, as shown by a significantly higher number and percentage of open arm entries, and a corresponding lower number of closed arm entries, as well as a greater percentage of time spent in the open arms (Figure 9).

To assess emotional memory, male and female DMSXL and WT littermate mice were tested in a fear conditioning paradigm*.* Both genotypes exhibited comparable freezing responses to a context or to a combined visual and auditory cue associated to a mild footshock, indicating a similar associative learning and memory (Figure 10A and B). However, when freezing behavior was analyzed in 1-min trial blocks, DMSXL mice showed significantly increased freezing time, in particular during the middle phase of the context test (Figure 10C, middle panel), suggesting an abnormal emotional response to the conditioned context. This heightened freezing response does not appear to be related to the muscular impairment of mutant mice, as the motor activity recorded in the testing chamber during the context session did not differ between genotypes, aside from a slight difference at minute 4 (Figure 10D, middle panel). Of note, DMSXL mice showed a reduced activity during the initial two minutes of the conditioning session possibly indicating early hypoactivity or altered response to a novel environment (Figure 10D, left panel).

### DMSXL mice show impaired sensorimotor gating and deficits in fine motor skills

At 15 weeks of age, sensorimotor gating and fine motor skills were assessed in WT and DMSXL homozygous mice using the acoustic startle response (ASR)/pre-pulse inhibition (PPI) test and the nest building assay, respectively. DMSXL mice exhibited a markedly reduced peak startle response to most of the 10 ms acoustic stimuli presented, as well as to the 110 dB, 40 ms startle pulse, compared to their wild-type littermates, (Figure 11A and B). Additionally, compared to wild-type littermates, DMSXL mice showed a statistically significant reduction of percentage pre-pulse inhibition indicating an impaired sensorimotor processing (Figure 11C and D). We can exclude auditory deficits in DMSXL mice since they retained the ability to inhibit their response to the startle pulse when it was preceded by a pre-pulse and all responded to a sudden click (20 kHz, 90 dB) eliciting a Preyer reflex (data not shown).

Fine motor abilities were assessed by providing singly housed mice with nesting material (cotton) and evaluating their capacity to construct a nest over a 48-h period. DMSXL mice of both sexes used significantly less cotton compared to their wild-type littermates (Figure 11E), indicating reduced forelimb skills. Consistently, nest quality scores were overall lower in DMSXL mice with a statistically significance difference only in females (Figure 11F).

### DMSXL mice show specific spatial and non-spatial memory deficits

To evaluate memory function, DMSXL and wild-type littermate mice were subjected to a working memory task (Y-maze) at the age of 14 weeks, and to a novel/displaced object recognition test, at the age of 20 weeks. As previously mentioned, the Y-maze assesses spatial working memory by measuring spontaneous alternation behavior. Moreover, the total number of entries can be used as a parameter of motor activity. DMSXL mice showed significantly fewer total arm entries compared to wild-type controls, suggesting reduced exploratory activity (Figure 12A), although no differences were found in the latency to exit the first arm (Figure 12B). Regarding the working memory, DMSXL mice showed a percentage of spontaneous alternation performance (SAP) similar to wild-types (Figure 12C), and a corresponding low percentage of alternate arm return (AAR, Figure 12D), indicating an intact working memory.

In the object recognition test, mice were habituated to two distinct objects placed in the open field arena over three 5-min sessions (S2-S4). In the subsequent session (S5), one of the familiar objects was relocated to a different position (displaced object), while in the last session (S6) the same object was replaced with a new one (novel object) (Supplementary Figure S4). In this task, while wild-type mice showed a significant preference for the displaced object over the non-displaced one, DMSXL mice failed to discriminate between objects, suggesting impaired spatial memory (Figure 12E). This finding was supported by the computation of the discrimination index (DI), calculated as the difference in time spent exploring the displaced object versus the familiar one, over the total exploration time in S5. DI was significantly lower in DMSXL mice compared to wild-type controls (Figure 12G). Moreover, wild-type mice exhibited a significant preference (p < 0.0001) for the displaced object, as shown by a DI significantly greater than zero whereas DMSXL mice showed a reduced but still significant discrimination (p < 0.05). In the novel object session (S6), wild-type mice spent significantly more time exploring the novel object compared to the familiar one whereas DMSXL mice did not show a significant preference, suggesting a deficit in novel object recognition (Figure 12F). This preference was not reflected in a DI significantly different from zero, both genotypes displaying comparable DI with no significant difference between groups (Figure 12H). This may indicate either high inter-individual variability or that the preference was not strong or consistent enough to be captured by the normalized index, highlighting the importance of using multiple complementary measures in object recognition tasks. Nevertheless, these CNR results are comparable to what was found in the French cohort at 4 months, despite differences in the test procedures.

## Discussion

In this study, we provide a comprehensive behavioral characterization of DMSXL transgenic mice carrying large expansions of CTG repeats in the human *DMPK* gene, using complementary longitudinal and cross-sectional approaches in two independent laboratories. Our results highlight a constellation of behavioral alterations reminiscent of the central nervous system (CNS) symptoms observed in DM1 patients, particularly those with early-onset forms of the disease.

Consistent with previous reports^54,87^ DMSXL mice displayed reduced body weight and muscle strength across both sexes, which, however, did not appear to interfere with the majority of behavioral outputs assessed. Importantly, longitudinal assessments revealed age-dependent changes in exploratory and anxiety-like behaviors, particularly in male mice. At early ages, DMSXL mice exhibited hypoactivity and novelty-induced inhibition, whereas from two months onwards, they showed increased center exploration in the open field. While longitudinal testing in Paris revealed increased center exploration in DMSXL males, indicative of reduced anxiety-like behavior, cross-sectional testing in Rome at 3 months showed reduced center time and fewer center entries in DMSXL males in the open field and reduced percentage of time in the light side of the L/D test, suggestive of heightened anxiety. Interestingly, in a previous study, we observed decreased vertical exploratory activity (percentage of rearing during the first minute of the initial 5 min of the first open-field session) and increased marble-burying, in concordance with novelty-induced inhibition and increased anxiety in response to novelty.^38^ Similarly, an altered response to novelty was observed in the first minutes of the fear conditioning test in DMSXL mice of the Italian cohort. Anxiety-related behaviors in rodents are known to be influenced by environmental conditions likely due to variations in experimental setup, lighting, arena size or material, and prior test experience. These aspects underscore the importance of contextual factors when interpreting behavioral data and highlight the need for standardized conditions in multicenter preclinical studies.^74^ Despite discrepancies, both sites consistently reported genotype-dependent alterations in emotional responses, reinforcing the translational value of the DMSXL model. Moreover, both laboratories found a greater time spent in open arms in the elevated plus maze, suggesting altered anxiety processing or impaired risk assessment. Such seemingly paradoxical behaviors across tests (open field and elevated plus maze) could be accounted for by differences in the underlying motivational and cognitive components: the open field mainly assesses exploratory drive, locomotor activity, and response to novelty, while the elevated plus maze is more dependent on conflict-based risk evaluation. Comparable paradoxical behavioral patterns have been reported in GLT1+/– mice, a model also showing reduced anxiety-related avoidance and impaired risk evaluation.^88^ Interestingly, previous work has shown decreased GLT1 expression in the cerebellum of DMSXL mice,^57^ suggesting a possible shared underlying mechanism. Importantly, these phenotypes were not confounded by hypoactivity, as locomotor performance was either preserved or increased in DMSXL males at later stages.

Cognitive assessments performed in the two laboratories revealed overall preserved working memory performance in the Y-maze test, with no genotype differences in alternation rates or initiation latency. Differences were observed around 3–4 months of age in the total number of arm entries between the two cohorts: the Italian cohort showed a reduced number of total arm entries in DMSXL mice compared to WT, while no such difference was detected in the French cohort at the same age. Reduced number of entries and total distance was however found in the French cohort at the 1-month Y-maze test, i.e., the first exposure to that context. Nevertheless, the discrepancy between cohorts may reflect differences in experimental environment (lighting, noise, odors, handling) as well as in habituation procedures or testing sequence, all of which are known to influence exploratory behavior in rodents. In addition, this test parameters have been already found suboptimal in achieving reproducibility across labs.^74^

Subtle impairments in long-term recognition memory emerged in the novel object recognition task at 4 months, where novelty preference was less robust and more variable in DMSXL mice from Paris. Similar results, despite different procedures, were obtained in the Italian cohort using a displaced object recognition task, further supporting a deficit in spatial novelty detection, consistent with the spatial memory impairment previously reported in the water maze test.^38^ These findings echo the visuo-spatial and executive impairments reported in DM1 patients^3,6,22,89^ and reinforce the notion that cognitive deficits in DMSXL mice are subtle, age-dependent, and task-specific. DMSXL mice also exhibited abnormalities in social behavior. While both genotypes showed a preference for the compartment containing a social stimulus during the sociability phase of the three-chamber test, the response was markedly attenuated in DMSXL mice. Furthermore, during the social novelty phase, only WT mice demonstrated a clear preference for the novel social partner, indicating a deficit in social discrimination in DMSXL animals. These results were supported by sniffing behavior analysis, although sniffing-based metrics appeared less sensitive to novelty detection. The combination of reduced sociability and impaired social recognition mirrors the social difficulties reported in pediatric DM1 patients, including autism spectrum-like features and impaired theory of mind.^8,17,^^90–92^

Additional and in-depth behavioral assessments from the Italian cohort further emphasized the CNS dysfunction in DMSXL mice. These included reduced night time voluntary wheel running activity in the home cage monitored 24/7, increased anxiety-related responses in the open field and light/dark tests, abnormal emotional responses in the fear conditioning task, and impaired sensorimotor gating in the PPI paradigm. Notably, DMSXL mice also showed reduced nest-building abilities, reflecting impaired fine motor coordination and reduced goal-directed behavior. Although DMSXL mice motor abilities impairments could reflect fatigue and deficits in motivation, we cannot exclude muscular involvement. DMSXL mice also show clear emotional dysfunctions and react abnormally to experimental contexts: impaired risk assessment and increased freezing could be connected to specific cognitive impairments such as abnormal sensorimotor processing and spatial memory deficits.

Together, these findings align with key neuropsychiatric and executive symptoms described in DM1 patients, including apathy, emotional dysregulation, attentional deficits, impaired initiative, fatigue and motor processing.^93^ The robust and reproducible alterations observed across two independent sites (findings are summarized in Table 1) strengthen the translational relevance of this model and underscore its potential for preclinical evaluation of CNS-targeted therapies. Beyond the identification of CNS-related phenotypes, this study also provides a detailed behavioral framework that can guide future therapeutic testing. By combining longitudinal and cross-sectional designs, we delineated a set of behavioral endpoints - such as altered anxiety-like behavior, impaired social discrimination, subtle cognitive impairment and sensorimotor gating deficits - that are both sensitive to genotype and relevant to patient symptoms. Importantly, our results help define the developmental trajectory of these phenotypes and highlight optimal time windows for preclinical studies during which therapeutic interventions may be most effective. This toolbox of behavioral assays thus constitutes a valuable resource for evaluating the efficacy of CNS-directed treatments in DM1, enabling both early detection of therapeutic benefit and long-term monitoring of disease progression. A potential limitation of this study is the frailty of DMSXL homozygous mice, which are known to display increased preweaning mortality and pose challenges for colony management and cohort production. In addition, these mice are homozygous knockout for the *Fbxl7* gene, into which the human *DMPK* transgene carrying large CTG expansions is inserted. Although *Fbxl7* knockout mice have not been reported to exhibit overt neurological or behavioral phenotypes (www.mousephenotype.org/data/genes/MGI:3052506), we cannot entirely exclude a contribution of this background. Nevertheless, several recent studies from our laboratory and others, focusing on the central nervous system, support the notion that the observed phenotypes are primarily driven by the presence of long CTG expansions and their downstream molecular consequences. (e.g.,^38,58,63,67^ rather than to the integration site. These findings support the interpretation that the behavioral alterations reported here predominantly reflect RNA toxicity and CNS dysfunction associated with repeat expansion.

**Table 1.** Comparative overview of behavioral assessments in DMSXL mice from the French (longitudinal) and Italian (cross-sectional) cohorts.

Behavioral domain	Test	Main parameters	Age(s) French cohort	Age(s) Italian cohort	French cohort (longitudinal)	Italian cohort (cross-sectional)	Comparison / interpretation	Figure
Locomotor / exploratory activity and novelty-induced inhibition	Open Field	Total distance, center time, center entries	1–12 months	3 months	No genotype effect on total distance; transient novelty-induced inhibition in females at 1 month, followed by normalization or increased center exploration in males at later ages	No genotype effect on total distance or average speed; reduced visits to center in DMSXL males and females during the first 5 min	Overall locomotor activity preserved in both cohorts. Both showed novelty-induced inhibition, transient in the French cohort and more pronounced in the italian cohort, likely reflecting environmental or procedural differences	Figures 3, 4, 7, 8
Anxiety / risk assessment	Elevated Plus Maze	Time & entries in open arms	1–4 months	3 months	↑ open-arm time at 4 months (↓ anxiety / disinhibition)	↑ open-arm entries & time (↓ avoidance)	Consistent altered risk assessment	Figures 4, 9
Anxiety	Light-Dark	Percentage time in light side	–	3 months	–	↓ % time in light side	Increased anxiety similar to open field % time and visits to center	Figures 8
Working memory	Y-maze	Alternation %, total entries	1–4 months	3 months	Preserved alternation; reduced locomotion at 1-month; normal locomotion 2–4 months	Fewer total entries; preserved alternation	Working memory preserved; locomotion not consistent at same age	Figures 5, 12
Recognition / spatial memory	Novel Object Recognition	Discrimination index, novelty preference	2–4 months	4.5 months	Subtle reduction of novelty preference at 4 months	Impaired discrimination for displaced object (↓ spatial memory)	Consistent mild cognitive deficit	Figures 5, 12
Social behavior	Three-chamber test	Sociability & social novelty preference	5 months	–	↓ sociability & ↓ social discrimination	–	Reduced sociability and social novelty preference	Figure 6
Sensorimotor gating	Acoustic startle response/prepulse inhibition	Startle amplitude, %PPI	–	3.5 months	–	↓ startle & ↓ PPI	Impaired startle response and sensorimotor gating	Figure 11
Motor skills / motivation	Nest building	Cotton used, nest score	–	3.5 months	–	↓ cotton use & lower nest quality	Fine motor & motivation deficits	Figure 11
Emotional learning	Fear conditioning	Freezing time	–	4 months	–	↑ freezing (abnormal emotional response)	Emotional dysregulation	Figure 10
Spontaneous activity	Running wheel	Distance, duration, speed	–	4 months	–	↓ voluntary running	Fatigue or reduced motivation	Figure 7

The table summarizes main parameters, ages tested, and key outcomes, highlighting both shared and site-specific findings across independent experimental settings.

In conclusion, we described a detailed behavioral profile of DMSXL mice that revealed CNS-relevant deficits strongly resembling the clinical phenotype of DM1 patients often observed in the severe forms of the disease. DM1 CNS-related symptoms such as fatigue, reduced processing speed, visuospatial impairments, blunted affect, social withdrawal, executive dysfunction, attention deficits, and apathy, have been associated with structural and functional brain abnormalities such as white matter lesions and altered frontostriatal and limbic connectivity.^94,95^ Such parallels not only support the translational validity of the DMSXL model for CNS-related pathology in DM1, but also highlight the potential of preclinical behavioral assays possibly combined to *in vivo* brain imaging to elucidate the mechanistic underpinnings of cognitive and affective dysfunction in this disorder.

## Supplemental Material

sj-pptx-1-jnd-10.1177_22143602251410998 - Supplemental material for Translational behavioral phenotypes in DMSXL mice for CNS manifestations of DM1 Supplemental material, sj-pptx-1-jnd-10.1177_22143602251410998 for Translational behavioral phenotypes in DMSXL mice for CNS manifestations of DM1 by Elisabetta Golini, Aline Huguet-Lachon, Hélène Benyamine, Noëmy Forasté Gueriba, Ferdinando Scavizzi, Marcello Raspa, Germana Falcone, Beatrice Cardinali, Silvia Mandillo and Geneviève Gourdon in Journal of Neuromuscular Diseases

## Abbreviations

DM1: Myotonic dystrophy type 1
CDM: Congenital myotonic dystrophy
*DMPK*: Dystrophia myotonica protein kinase
MBNL: Muscleblind-like Protein
CELF: CUGBP Elav-like Family Member
CNS: central nervous system

## References

[1] Harper PS . Myotonic dystrophy, 3rd edition. London: WB Saunders, 2001.

[2] Meola G . Myotonic dystrophies as a brain disorder. Neurol Sci 2010; 31: 863–864. 20924632 10.1007/s10072-010-0414-2

[3] Gourdon G Meola G . Myotonic dystrophies: state of the art of new therapeutic developments for the CNS. Front Cell Neurosci 2017; 11: 1–14. 28473756 10.3389/fncel.2017.00101 PMC5397409

[4] Weijs R Okkersen K van Engelen B , et al. Human brain pathology in myotonic dystrophy type 1: a systematic review. Neuropathology 2021; 41: 3–20. 33599033 10.1111/neup.12721 PMC7986875

[5] Angeard N Gargiulo M Jacquette A , et al. Cognitive profile in childhood myotonic dystrophy type 1: is there a global impairment? Neuromuscul Disord 2007; 17: 451–458. 17433680 10.1016/j.nmd.2007.02.012

[6] Angeard N Jacquette A Gargiulo M , et al. A new window on neurocognitive dysfunction in the childhood form of myotonic dystrophy type 1 (DM1). Neuromuscul Disord 2011; 21: 468–476. 21592796 10.1016/j.nmd.2011.04.009

[7] Meola G Sansone V . Cerebral involvement in myotonic dystrophies. Muscle Nerve 2007; 36: 294–306. 17486579 10.1002/mus.20800

[8] Ekström AB Hakenäs-Plate L Samuelsson L , et al. Autism spectrum conditons in myotonic dystrophy type 1: a study on 57 individuals with congenital and childhood forms. Am J Med Genet Part B Neuropsychiatr Genet 2008; 147: 918–926. 10.1002/ajmg.b.30698 18228241

[9] Ekström AB Hakenäs-Plate LO Tulinius M , et al. Cognition and adaptive skills in myotonic dystrophy type 1: a study of 55 individuals with congenital and childhood forms. Dev Med Child Neurol 2009; 51: 982–990. 19459914 10.1111/j.1469-8749.2009.03300.x

[10] Douniol M Jacquette A Guilé JM , et al. Psychiatric and cognitive phenotype in children and adolescents with myotonic dystrophy. Eur Child Adolesc Psychiatry 2009; 18: 705–715. 19543792 10.1007/s00787-009-0037-4

[11] Douniol M Jacquette A Cohen D , et al. Psychiatric and cognitive phenotype of childhood myotonic dystrophy type 1. Dev Med Child Neurol 2012; 54: 905–911. 22861906 10.1111/j.1469-8749.2012.04379.x

[12] Johnson NE Luebbe E Eastwood E , et al. The impact of congenital and childhood myotonic dystrophy on quality of life: a qualitative study of associated symptoms. J Child Neurol 2014; 29: 983–986. 23611887 10.1177/0883073813484804

[13] Jean S Richer L Laberge L , et al. Comparisons of intellectual capacities between mild and classic adult-onset phenotypes of myotonic dystrophy type 1 (DM1). Orphanet J Rare Dis 2014; 9: 86. 25424323 10.1186/s13023-014-0186-5 PMC4247010

[14] Rubinsztein JS Rubinsztein DC Goodburn S , et al. Apathy and hypersomnia are common features of myotonic dystrophy. J Neurol Neurosurg Psychiatry 1998; 64: 510–515. 9576545 10.1136/jnnp.64.4.510 PMC2170039

[15] Modoni A Silvestri G Pomponi MG , et al. Characterization of the pattern of cognitive impairment in myotonic dystrophy type 1. Arch Neurol 2004; 61: 1943–1947. 15596617 10.1001/archneur.61.12.1943

[16] Sansone V Gandossini S Cotelli M , et al. Cognitive impairment in adult myotonic dystrophies: a longitudinal study. Neurol Sci 2007; 28: 9–15. 17385090 10.1007/s10072-007-0742-z

[17] Leddy S Cercignani M Serra L , et al. Social cognition in type 1 myotonic dystrophy – A mini review. Cortex 2021; 142: 389–399. 34154799 10.1016/j.cortex.2021.05.004

[18] Gallais B Montreuil M Gargiulo M , et al. Prevalence and correlates of apathy in myotonic dystrophy type 1. BMC Neurol 2015; 15: 48. 26296336 10.1186/s12883-015-0401-6 PMC4546188

[19] Takeda A Kobayakawa M Suzuki A , et al. Lowered sensitivity to facial emotions in myotonic dystrophy type 1. J Neurol Sci 2009; 280: 35–39. 19223261 10.1016/j.jns.2009.01.014

[20] Kobayakawa M Tsuruya N Takeda A , et al. Facial emotion recognition and cerebral white matter lesions in myotonic dystrophy type 1. J Neurol Sci 2010; 290: 48–51. 20006353 10.1016/j.jns.2009.11.011

[21] Masaoka Y Kawamura M Takeda A , et al. Impairment of odor recognition and odor-induced emotions in type 1 myotonic dystrophy. Neurosci Lett 2011; 503: 163–166. 21884754 10.1016/j.neulet.2011.08.006

[22] Winblad S Samuelsson L Lindberg C , et al. Cognition in myotonic dystrophy type 1: a 5-year follow-up study. Eur J Neurol 2016; 23: –6. 10.1111/ene.13062 27323306

[23] Laberge L Begin P Dauvilliers Y , et al. A polysomnographic study of daytime sleepiness in myotonic dystrophy type 1. J Neurol Neurosurg Psychiatry 2009; 80: 642–646. 19211594 10.1136/jnnp.2008.165035

[24] Heatwole C Bode R Johnson N , et al. Patient-reported impact of symptoms in myotonic dystrophy type 1 (PRISM-1). Neurology 2012; 79: 348–357. 22786587 10.1212/WNL.0b013e318260cbe6 PMC3400095

[25] Laberge L Gagnon C Dauvilliers Y . Daytime sleepiness and myotonic dystrophy. Curr Neurol Neurosci Rep 2013; 13: 40. 10.1007/s11910-013-0340-9 23430686

[26] Angelini C Tasca E . Fatigue in muscular dystrophies. Neuromuscul Disord 2012; 22: 214–220. 10.1016/j.nmd.2012.10.010 PMC3526799 23182642

[27] Harper PS . Myotonic dystrophy, 3rd Edition - Major problems in neurology. Oxford: OUP, 2009.

[28] Sicot G Gourdon G Gomes-Pereira M . Myotonic dystrophy, when simple repeats reveal complex pathogenic entities: new findings and future challenges. Hum Mol Genet 2011; 20: R116–R123. 10.1093/hmg/ddr343 21821673

[29] Nemoto J Nakamori M . Molecular genetics of myotonic dystrophy and the evolution of therapeutic approaches. J Hum Genet 2025. DOI: 10.1038/s10038-025-01358-6. 40603638

[30] Jiang H Mankodi A Swanson MS , et al. Myotonic dystrophy type 1 is associated with nuclear foci of mutant RNA, sequestration of muscleblind proteins and deregulated alternative splicing in neurons. Hum Mol Genet 2004; 13: 3079–3088. 15496431 10.1093/hmg/ddh327

[31] Caillet-Boudin M-L Fernandez-Gomez F-J Tran H , et al. Brain pathology in myotonic dystrophy: when tauopathy meets spliceopathy and RNAopathy. Front Mol Neurosci 2014; 6: 57. 24409116 10.3389/fnmol.2013.00057 PMC3885824

[32] Nishi M Kimura T Furuta M , et al. Differences in splicing defects between the grey and white matter in myotonic dystrophy type 1 patients. Plos ONE 2020. DOI: 10.1371/journal.pone.0224912. PMC7224547 32407311

[33] Degener MJF Van Cruchten RTP Otero BA , et al. A comprehensive atlas of fetal splicing patterns in the brain of adult myotonic dystrophy type 1 patients. NAR Genom Bioinform 2022; 4. DOI: 10.1093/nargab/lqac016. PMC8903011 35274098

[34] Yoshizumi K Nishi M Igeta M , et al. Analysis of splicing abnormalities in the white matter of myotonic dystrophy type 1 brain using RNA sequencing. Neurosci Res 2024; 200: 48–56. 37806497 10.1016/j.neures.2023.10.002

[35] Wojciechowska M Srinivasan A Magner D , et al. Global dysregulation of circular RNAs in frontal cortex and whole blood from DM1 and DM2. Original Investigation Human Genetics 2025; 144: 417–432. 10.1007/s00439-025-02729-x PMC12003446 39903274

[36] Charizanis K Lee KY Batra R , et al. Muscleblind-Like 2 mediated alternative splicing in the developing brain and dysregulation in myotonic dystrophy. Neuron 2012; 75: 437–450. 22884328 10.1016/j.neuron.2012.05.029 PMC3418517

[37] Goodwin M Mohan A Batra R , et al. MBNL Sequestration by toxic RNAs and RNA misprocessing in the myotonic dystrophy brain. Cell Rep 2015; 12: 1159–1168. 26257173 10.1016/j.celrep.2015.07.029 PMC4545389

[38] Hernandez-Hernandez O Guiraud-Dogan C Sicot G , et al. Myotonic dystrophy CTG expansion affects synaptic vesicle proteins, neurotransmission and mouse behaviour. Brain 2013; 136: 957–970. 23404338 10.1093/brain/aws367 PMC3580270

[39] Ladd AN . CUG-BP, elav-like family (CELF)-mediated alternative splicing regulation in the brain during health and disease. Mol Cell Neurosci 2013; 56: 456–464. 23247071 10.1016/j.mcn.2012.12.003 PMC3650117

[40] Jiang H Mankodi A Swanson MS , et al. Myotonic dystrophy type 1 is associated with nuclear foci of mutant RNA, sequestration of muscleblind proteins and deregulated alternative splicing in neurons. Hum Mol Genet 2004; 13: 3079–3088. 15496431 10.1093/hmg/ddh327

[41] Otero BA Poukalov K Hildebrandt RP , et al. Transcriptome alterations in myotonic dystrophy frontal cortex. Cell Rep 2021; 34. DOI: 10.1016/j.celrep.2020.108634. PMC9272850 33472074

[42] Azotla-Vilchis CN Sanchez-Celis D Agonizantes-Juárez LE , et al. Transcriptome analysis reveals altered inflammatory pathway in an inducible glial cell model of myotonic dystrophy type 1. Biomolecules 2021; 11. DOI: 10.3390/biom11020159. PMC7910866 33530452

[43] Nakamori M Shimizu H Ogawa K , et al. Cell type-specific abnormalities of central nervous system in myotonic dystrophy type 1. Brain Commun 2022; 4. DOI: 10.1093/braincomms/fcac154. PMC9218787 35770133

[44] Nutter CA Kidd BM Carter HA , et al. Choroid plexus mis-splicing and altered cerebrospinal fluid composition in myotonic dystrophy type 1. Brain 2023; 146. DOI: 10.1093/brain/awad148. PMC10545633 37143315

[45] Edokpolor KS Banerjee A McEachin ZT , et al. Altered behavioral responses show GABA sensitivity in muscleblind-like 2-deficient mice: implications for CNS symptoms in myotonic dystrophy. eNeuro 2022; 9. DOI: 10.1523/ENEURO.0218-22.2022. PMC9557336 36150891

[46] Wang PY Lin YM Wang LH , et al. Reduced cytoplasmic MBNL1 is an early event in a brain-specific mouse model of myotonic dystrophy. Hum Mol Genet 2017; 26. DOI: 10.1093/hmg/ddx115. 28369378

[47] Lee KY Chang HC Seah C , et al. Deprivation of muscleblind-like proteins causes deficits in cortical neuron distribution and morphological changes in dendritic spines and postsynaptic densities. Front Neuroanat 2019; 13. DOI: 10.3389/fnana.2019.00075. PMC6682673 31417371

[48] Ramon-Duaso C Rodríguez-Morató J Selma-Soriano E , et al. Protective effects of mirtazapine in mice lacking the Mbnl2 gene in forebrain glutamatergic neurons: Relevance for myotonic dystrophy 1. Neuropharmacology 2020; 170. DOI: 10.1016/j.neuropharm.2020.108030. 32171677

[49] Ramon-Duaso C Gener T Consegal M , et al. Methylphenidate attenuates the cognitive and mood alterations observed in Mbnl2 knockout mice and reduces microglia overexpression. Cereb Cortex 2019; 29. DOI: 10.1093/cercor/bhy164. PMC7963113 30060068

[50] Matynia A Ng CH Dansithong W , et al. Muscleblind1, but not Dmpk or Six5, contributes to a complex phenotype of muscular and motivational deficits in mouse models of Myotonic Dystrophy. PLoS One 2010; 5: 1–9. 10.1371/journal.pone.0009857 PMC2845609 20360842

[51] Sznajder L Khan M Tadross M , et al. Autistic traits in myotonic dystrophy type 1 due to MBNL inhibition and RNA mis-splicing. Res Sq 2023. DOI: 10.21203/rs.3.rs-3221704/v1.

[52] Seznec H Agbulut O Sergeant N , et al. Mice transgenic for the human myotonic dystrophy region with expanded CTG repeats display muscular and brain abnormalities. Hum Mol Genet 2001; 10: 2717–2726. 11726559 10.1093/hmg/10.23.2717

[53] Gomes-Pereira M Foiry L Nicole A , et al. CTG Trinucleotide repeat ‘big jumps’: large expansions, small mice. PLoS Genet 2007; 3: 52. 10.1371/journal.pgen.0030052 PMC1847694 17411343

[54] Huguet A Medja F Nicole A , et al. Molecular, physiological, and motor performance defects in DMSXL mice carrying >1,000 CTG repeats from the human DM1 locus. PLoS Genet 2012; 8: e1003043. 10.1371/journal.pgen.1003043 PMC3510028 23209425

[55] Michel L Huguet-Lachon A Gourdon G . Sense and antisense DMPK RNA foci accumulate in DM1 tissues during development. PLoS One 2015; 10: 1–17. 10.1371/journal.pone.0137620 PMC4560382 26339785

[56] González-Barriga A Lallemant L Dincã DM , et al. Integrative cell type-specific multi-omics approaches reveal impaired programs of glial cell differentiation in mouse culture models of DM1. Front Cell Neurosci 2021; 15. DOI: 10.3389/fncel.2021.662035. PMC8136287 34025359

[57] Sicot G Servais L Dinca DM , et al. Downregulation of the glial GLT1 glutamate transporter and purkinje cell dysfunction in a mouse model of myotonic dystrophy. Cell Rep 2017; 19: 2718–2729. 28658620 10.1016/j.celrep.2017.06.006 PMC8496958

[58] Dincã DM Lallemant L González-Barriga A , et al. Myotonic dystrophy RNA toxicity alters morphology, adhesion and migration of mouse and human astrocytes. Nat Commun 2022; 13. DOI: 10.1038/s41467-022-31594-9. PMC9253038 35789154

[59] Golini E Rigamonti M Raspa M , et al. Excessive rest time during active phase is reliably detected in a mouse model of myotonic dystrophy type 1 using home cage monitoring. Front Behav Neurosci 2023; 17. DOI: 10.3389/fnbeh.2023.1130055. PMC10017452 36935893

[60] Nieuwenhuis S Kozakov D Kapusta K , et al. Altered reversal and extinction learning in the DMSXL mouse model of type I myotonic dystrophy (DM1): an exploratory study. J Neuromuscul Dis 2025; 12: 535–547. 40400327 10.1177/22143602251339350 PMC13142853

[61] Potier B Lallemant L Parrot S , et al. DM1 Transgenic mice exhibit abnormal neurotransmitter homeostasis and synaptic plasticity in association with RNA foci and mis-splicing in the hippocampus. Int J Mol Sci 2022; 23: 92. 10.3390/ijms23020592 PMC8775431 35054778

[62] Lutz M Levanti M Karns R , et al. Therapeutic targeting of the GSK3β-CUGBP1 pathway in myotonic dystrophy. Int J Mol Sci 2023; 2023: 10650. 10.3390/ijms241310650 PMC10342152 37445828

[63] Ait Benichou S Jauvin D De Serres-Bérard T , et al. Enhanced delivery of ligand-conjugated antisense oligonucleotides (C16-HA-ASO) targeting dystrophia myotonica protein kinase transcripts for the treatment of myotonic dystrophy type 1. Hum Gene Ther 2022; 33. DOI: 10.1089/hum.2022.069. 35794764

[64] Ait Benichou S Jauvin D De Serres-Bérard T , et al. Antisense oligonucleotides as a potential treatment for brain deficits observed in myotonic dystrophy type 1. Gene Ther 2022; 29: 698–709. 35075265 10.1038/s41434-022-00316-7 PMC9750879

[65] Cardinali B Provenzano C Izzo M , et al. Time-controlled and muscle-specific CRISPR/Cas9-mediated deletion of CTG-repeat expansion in the DMPK gene. Mol Ther Nucleic Acids 2022; 27. DOI: 10.1016/j.omtn.2021.11.024. PMC8693309 34976437

[66] Izzo M Battistini J Golini E , et al. Muscle-specific gene editing improves molecular and phenotypic defects in a mouse model of myotonic dystrophy type 1. Clin Transl Med 2025. DOI: 10.1002/ctm2.70227. PMC11830570 39956955

[67] Wang M Weng W-C Stock L , et al. Correction of glycogen synthase kinase 3 β in myotonic dystrophy 1 reduces the mutant RNA and improves postnatal survival of DMSXL mice. Mol Cell Biol 2019; 39. DOI: 10.1128/mcb.00155-19. PMC6791656 31383751

[68] Lo Scrudato M Poulard K Sourd C , et al. Genome editing of expanded CTG repeats within the human DMPK gene reduces nuclear RNA foci in the muscle of DM1 mice. Mol Ther 2019; 27: 1372–1388. 31253581 10.1016/j.ymthe.2019.05.021 PMC6697452

[69] Jauvin D Chrétien J Pandey SK , et al. Targeting DMPK with antisense oligonucleotide improves muscle strength in myotonic dystrophy type 1 mice. Mol Ther Nucleic Acids 2017; 7: 465–474. 28624222 10.1016/j.omtn.2017.05.007 PMC5453865

[70] Seznec H Lia-Baldini AS Duros C , et al. Transgenic mice carrying large human genomic sequences with expanded CTG repeat mimic closely the DM CTG repeat intergenerational and somatic instability. Hum Mol Genet 2000; 9: 1185–1194. 10767343 10.1093/hmg/9.8.1185

[71] Leger M Quiedeville A Bouet V , et al. Object recognition test in mice. Nat Protoc 2013; 8. DOI: 10.1038/nprot.2013.155. 24263092

[72] Antunes M Biala G . The novel object recognition memory: neurobiology, test procedure, and its modifications. Cogn Process 2012; 13. DOI: 10.1007/s10339-011-0430-z. PMC3332351 22160349

[73] Provenzano C Cappella M Valaperta R , et al. CRISPR/Cas9-mediated deletion of CTG expansions recovers normal phenotype in myogenic cells derived from myotonic dystrophy 1 patients. Mol Ther Nucleic Acids 2017; 9. DOI: 10.1016/j.omtn.2017.10.006. PMC5684470 29246312

[74] Mandillo S Tucci V Hölter SM , et al. Reliability, robustness, and reproducibility in mouse behavioral phenotyping: a cross-laboratory study. Physiol Genomics 2008; 34: 243–255. 18505770 10.1152/physiolgenomics.90207.2008 PMC2519962

[75] Carola V D’Olimpio F Brunamonti E , et al. Evaluation of the elevated plus-maze and open-field tests for the assessment of anxiety-related behaviour in inbred mice. Behav Brain Res 2002; 134. DOI: 10.1016/S0166-4328(01)00452-1. 12191791

[76] Seibenhener ML Wooten MC . Use of the open field maze to measure locomotor and anxiety-like behavior in mice. J Visualized Exp 2015. DOI: 10.3791/52434. PMC4354627 25742564

[77] Mandillo S Heise I Garbugino L , et al. Early motor deficits in mouse disease models are reliably uncovered using an automated home-cage wheel-running system: A cross-laboratory validation. DMM Dis Models Mech 2014; 7. DOI: 10.1242/dmm.013946. PMC3944499 24423792

[78] Crawley J Goodwin FK . Preliminary report of a simple animal behavior model for the anxiolytic effects of benzodiazepines. Pharmacol Biochem Behav 1980; 13. DOI: 10.1016/0091-3057(80)90067-2. 6106204

[79] Mandillo S Golini E Marazziti D , et al. Mice lacking the Parkinson’s related GPR37/PAEL receptor show non-motor behavioral phenotypes: age and gender effect. Genes Brain Behav 2013; 12. DOI: 10.1111/gbb.12041. 23574697

[80] Lister RG . The use of a plus-maze to measure anxiety in the mouse. Psychopharmacology 1987; 92. DOI: 10.1007/BF00177912. 3110839

[81] Bitetti A Mallory AC Golini E , et al. MicroRNA degradation by a conserved target RNA regulates animal behavior. Nat Struct Mol Biol 2018; 25. DOI: 10.1038/s41594-018-0032-x. 29483647

[82] Rustay N Browman K Curzon P . Cued and contextual fear conditioning for rodents. Front Neurosci 2008; 2008. DOI: 10.1201/noe1420052343.ch2. 21204331

[83] Swerdlow NR Geyer MA . Using an animal model of deficient sensorimotor gating to study the pathophysiology and new treatments of schizophrenia. Schizophr Bull 1998; 24. DOI: 10.1093/oxfordjournals.schbul.a033326. 9613626

[84] Deacon RMJ . Assessing nest building in mice. Nat Protoc 2006; 1. DOI: 10.1038/nprot.2006.170. 17406392

[85] Mandillo S Rinaldi A Oliverio A , et al. Repeated administration of phencyclidine, amphetamine and MK-801 selectively impairs spatial learning in mice: A possible model of psychotomimetic drug-induced cognitive deficits. Behav Pharmacol 2003; 14. DOI: 10.1097/00008877-200311000-00006. 14557721

[86] Branchi I Ricceri L . Scoring learning and memory in developing rodents. Curr Protoc Toxicol 2006; 13. DOI: 10.1002/0471140856.tx1311s27. 23045126

[87] Decostre V Vignaud A Matot B , et al. Longitudinal in vivo muscle function analysis of the DMSXL mouse model of myotonic dystrophy type 1. Neuromuscul Disord 2013; 23: 1016–1025. 24139022 10.1016/j.nmd.2013.07.014

[88] Kiryk A Aida T Tanaka K , et al. Behavioral characterization of GLT1 (+/-) mice as a model of mild glutamatergic hyperfunction. Neurotox Res 2008; 13. DOI: 10.1007/BF03033364. 18367437

[89] Gallais B Gagnon C Mathieu J , et al. Cognitive decline over time in adults with myotonic dystrophy type 1: A 9-year longitudinal study. Neuromuscul Disord 2016. DOI: 10.1016/j.nmd.2016.10.003. 27919548

[90] Angeard N Huerta E Jacquette A , et al. Childhood-onset form of myotonic dystrophy type 1 and autism spectrum disorder: is there comorbidity? Neuromuscul Disord 2018; 28: 216–221. 29361396 10.1016/j.nmd.2017.12.006

[91] Kobayakawa M Tsuruya N Kawamura M . Theory of mind impairment in adult-onset myotonic dystrophy type 1. Neurosci Res 2012; 72: 341–346. 22326781 10.1016/j.neures.2012.01.005

[92] Davion JB Tard C Kuchcinski G , et al. Characterization of theory of mind performance in patients with myotonic dystrophy type 1. Cortex 2023; 168. DOI: 10.1016/j.cortex.2023.07.008. 37742438

[93] Park JS Seo J Cha H , et al. Altered power spectral density in the resting-state sensorimotor network in patients with myotonic dystrophy type. Sci Rep 2018; 8. DOI: 10.1038/s41598-018-19217-0. PMC5772436 29343751

[94] Okkersen K Monckton DG Le N , et al. Brain imaging in myotonic dystrophy type 1: a systematic review. Neurology 2017; 89. 10.1212/WNL.0000000000004300 28768849

[95] Wu Y Wei Q Lin J , et al. Cognitive impairment, neuroimaging abnormalities, and their correlations in myotonic dystrophy: a comprehensive review. Front Cell Neurosci 2024; 18. DOI: 10.3389/fncel.2024.1369332. PMC11024338 38638300

